# Hypoxia-inducible factor prolyl-4-hydroxylase-1 is a convergent point in the reciprocal negative regulation of NF-κB and p53 signaling pathways

**DOI:** 10.1038/s41598-017-17376-0

**Published:** 2017-12-08

**Authors:** Karim Ullah, Ann-Helen Rosendahl, Valerio Izzi, Ulrich Bergmann, Taina Pihlajaniemi, Joni M. Mäki, Johanna Myllyharju

**Affiliations:** 1 Oulu Center for Cell-Matrix Research, University of Oulu, Oulu, FIN-90014 Finland; 2 Biocenter Oulu, University of Oulu, Oulu, FIN-90014 Finland; 3 Faculty of Biochemistry and Molecular Medicine, University of Oulu, Oulu, FIN-90014 Finland

## Abstract

Hypoxia-inducible factor 1α (HIF1α) induces the expression of several hundred genes in hypoxia aiming at restoration of oxygen homeostasis. HIF prolyl-4-hydroxylases (HIF-P4Hs) regulate the stability of HIF1α in an oxygen-dependent manner. Hypoxia is a common feature in inflammation and cancer and the HIF pathway is closely linked with the inflammatory NF-κB and tumor suppressor p53 pathways. Here we show that genetic inactivation or chemical inhibition of HIF-P4H-1 leads to downregulation of proinflammatory genes, while proapoptotic genes are upregulated. HIF-P4H-1 inactivation reduces the inflammatory response under LPS stimulus *in vitro* and in an acute skin inflammation model *in vivo*. Furthermore, HIF-P4H-1 inactivation increases p53 activity and stability and hydroxylation of proline 142 in p53 has an important role in this regulation. Altogether, our data suggest that HIF-P4H-1 inhibition may be a promising therapeutic candidate for inflammatory diseases and cancer, enhancing the reciprocal negative regulation of the NF-κB and p53 pathways.

## Introduction

Under compromized oxygenation, a hypoxia response pathway is induced by hypoxia-inducible factors (HIFs) that activate >100 genes for hypoxic adaptation^1,2^. HIF is an αβ dimer that accumulates in hypoxia, the α subunit being degraded in normoxia. HIF prolyl-4-hydroxylases (HIF-P4Hs 1–3, also known as PHDs 1–3 and EGLN 2, 1 and 3, respectively), the activity of which depends on molecular oxygen, iron and 2-oxoglutarate^3–9^, act as the oxygen sensors. HIF-P4Hs hydroxylate HIF1α in normoxia, leading to its ubiquitination and proteasomal degradation. Hypoxia inhibits HIF-P4Hs resulting in HIF1α stabilization and assembly of active HIF1 αβ dimer.

HIF is stabilized in resident inflamed cells and infiltrating immune cells that become exposed to hypoxia in active inflammation^10–13^. NF-κB is the key regulator of inflammatory genes and is activated by various inflammatory stimuli^14,15^. Extensive crosstalk exists between the hypoxia and NF-κB signaling pathways^11–13^. Studies using mainly siRNA approaches have indicated that HIF-P4H-1 affects NF-κB signaling at the post-translational level, but the exact targets of proline hydroxylation in the pathway remain to be identified^16–19^. Furthermore, *in vivo* mouse studies have shown that HIF-P4H inhibitors and genetic *Hif-p4h-1* and *Hif-p4h-3* inactivation have protective effects in inflammatory mucosal inflammation, involving reduced inflammatory cytokine production and epithelial cell apoptosis, increased re-epithelialization of colonic wounds and regulation of hypoxic neutrophil survival^20–26^.

In addition to the interplay of hypoxia and inflammation pathways, the tumor suppressor p53 affects both of them. Hypoxia-induced increase in p53 mRNA, translation, protein stability and activity, and decreased proteasomal p53 degradation, have been reported^27,28^. p53 is present at low baseline levels in a complex with MDM2 that blocks p53 activity and directs it to ubiquitination and proteasomal degradation, but p53 is stabilized and activated in response to DNA damage and other adverse stimuli^29^. MDM2-mediated interaction between p53 and HIF1α affecting their stability and activity has been reported^27,30^. NF-κB and p53 signaling are interconnected by reciprocal negative regulation and p53 is regarded as a negative regulator of inflammation^31–33^. HIF-P4H-1 has recently been linked to p53 regulation, silencing of HIF-P4H-1 increasing chemotherapy effectiveness in colorectal cancer cell lines via prevention of p53 activation, inhibition of DNA repair and increased cell death^34^.

To study the potential of HIF-P4H-1 being a common regulator of hypoxia, inflammatory and p53 pathways, we analyzed gene expression in *Hif-p4h-1* ^−/−^ mouse embryonic fibroblasts (MEFs) and found that proinflammatory genes were downregulated, while proapoptotic genes were upregulated. The inflammatory response was suppressed in *Hif-p4h-1* ^−/−^ MEFs and macrophages and in other cell types where HIF-P4H-1 was silenced, involving NF-κB downregulation. Furthermore, p53 stability was increased in HIF-P4H-1 deficient cells and hydroxylation of p53 proline 142 had a role in this. Finally, *Hif-p4h-1* ^−/−^ mice had a reduced acute skin inflammation, as well as an increased apoptotic activity in the skin. Altogether, our data suggest that HIF-P4H-1 inhibition enhances reciprocal negative regulation of the NF-κB and p53 pathways and may be a promising therapeutic candidate for inflammatory diseases and cancer.

## Results

### Expression of inflammatory and apoptotic genes is altered in *Hif-p4h-1*^−/−^ MEFs

**Figure 1:**
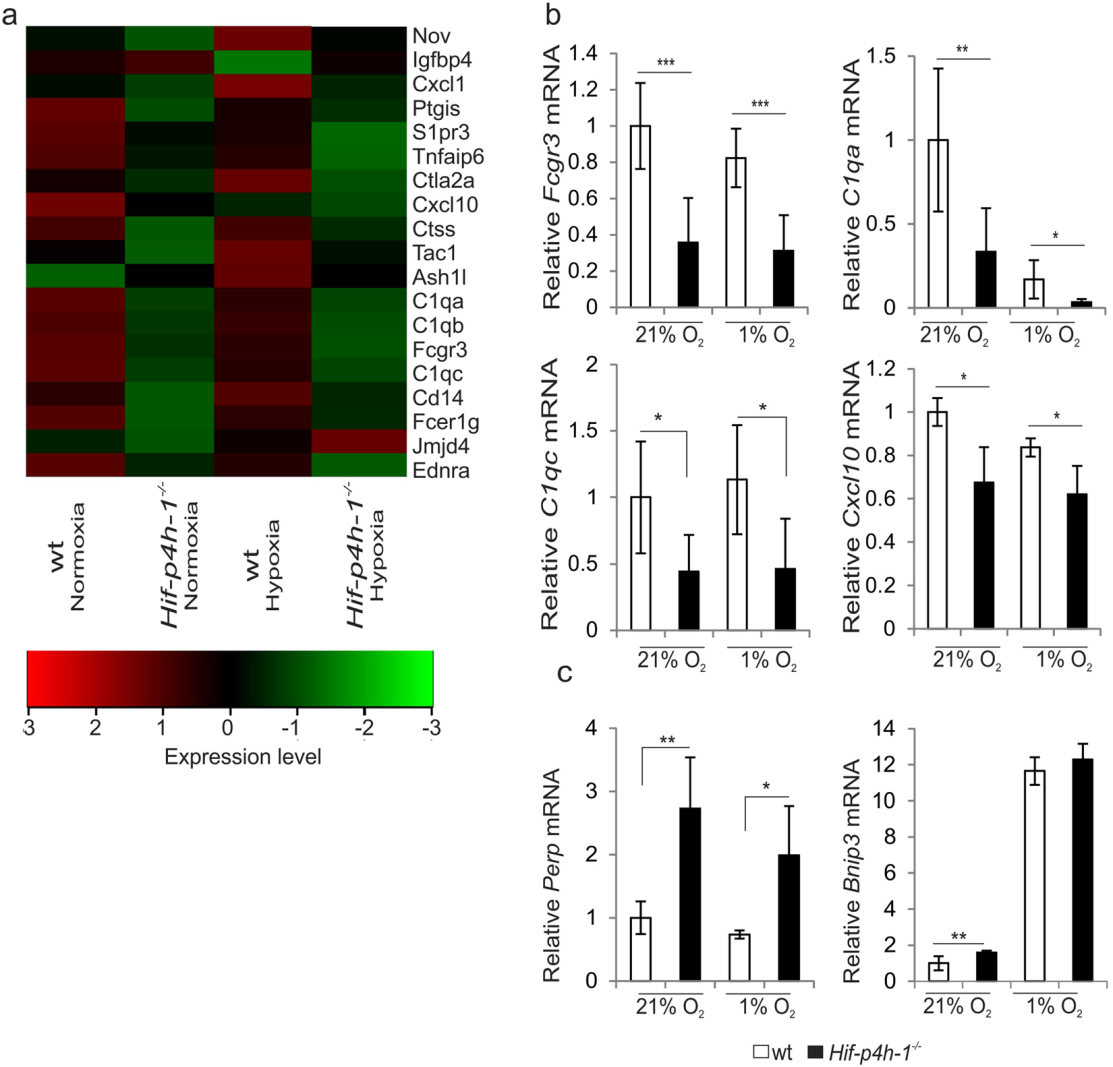
Lack of HIF-P4H-1 affects the expression of several genes involved in inflammatory and apoptotic pathways. (**a**) Heatmap analysis of microarray data shows that expression of several genes involved in proinflammatory pathways is downregulated in *Hif-p4h-1* ^−/−^ MEFs relative to wt after 24 h culture in normoxia (21% O_2_) and hypoxia (1% O_2_). A comparison between one set of samples is shown in the heatmap. The heatmap was generated by Chipster using Pearson correlation as a distance measure and average linkage for constructing the dendrogram. The red color represents upregulated genes and the green color represents downregulated genes. The values in the color key represent the expression levels between the samples. (**b**) qPCR analysis verifies that the expression of genes for the low affinity III receptor of the Fc fragment of IgG (*Fcgr3*), the α and γ chains of the complement C1q subcomponent (*C1qa*, *C1qc*) and the C-X-C motif chemokine 10 (*Cxcl10*) is downregulated in *Hif-p4h-1* ^−/−^ MEFs relative to wt in normoxia and hypoxia. (**c**) qPCR analysis shows that expression of the pro-apoptotic genes for the p53 apoptosis effector related to peripheral myelin protein 22 (*Perp*) and BCL2/adenovirus E1B 19 kDa protein-interacting protein 3 (*Bnip3*) is upregulated in *Hif-p4h-1* ^−/−^ MEFs relative to wt. (**b**,**c**) data are presented as mean ± s.d., n = 3–4 individual *Hif-p4h-1* ^−/−^ and wt MEF isolates analysed in triplicates. *P < 0.05, **P < 0.01 and ***P < 0.001, two-tailed Student’s *t*-test.

We performed microarray analysis of normoxic and hypoxic *Hif-p4h-1* ^−/−^ and wild-type (wt) MEFs isolated from a *Hif-p4h-1* ^−/−^ mouse line (supplementary Fig. 1). Analysis of the microarray data by dChip software showed that a total of 1093 genes were differentially expressed in *Hif-p4h-1* ^−/−^ and wt MEFs. Of these ~2.7% were genes that are involved in positive regulation of acute inflammatory response and 72.5% of these were downregulated in *Hif-p4h-1* ^−/−^ MEFs relative to wt (Fig. 1a, supplementary Table 1). Approximately 5.7% of the differentially expressed genes are involved in the regulation of apoptosis. 56% of these genes are involved in positive regulation of apoptosis, while 44% are classified as anti-apoptotic (supplementary Tables 2 and 3). Generally, antiapoptotic genes were slightly downregulated in *Hif-p4h-1* ^−/−^ MEFs, whereas for example *Perp*, a gene induced in p53-mediated apoptosis^35^, was upregulated (supplementary Tables 2 and 3).

Quantitative real-time PCR (qRT-PCR) confirmed the effect on selected inflammatory genes, for example, the *Fcgr3*, *C1qa*, *C1qc* and *Cxcl10* mRNA levels were decreased by 26–52% in *Hif-p4h-1* ^−/−^ MEFs relative to wt in normoxia and hypoxia (Fig. 1b). The mRNA level of *Perp* and another proapoptotic gene *Bnip3* was increased to 270% and 180%, respectively, in *Hif-p4h-1* ^−/−^ MEFs relative to wt in normoxia (Fig. 1c). A similar difference was seen in the *Perp* mRNA level between the genotypes in hypoxia (Fig. 1c). The *Bnip3* mRNA was highly induced in hypoxia in both *Hif-p4h-1* ^−/−^ and wt MEFs, but the trend of higher expression level in *Hif-p4h-1* ^−/−^ MEFs relative to wt was not statistically significant (Fig. 1c). The data indicate that HIF-P4H-1 affects the regulation of inflammatory and apoptotic genes.

### Lack of HIF-P4H-1 suppresses inflammatory response

**Figure 2:**
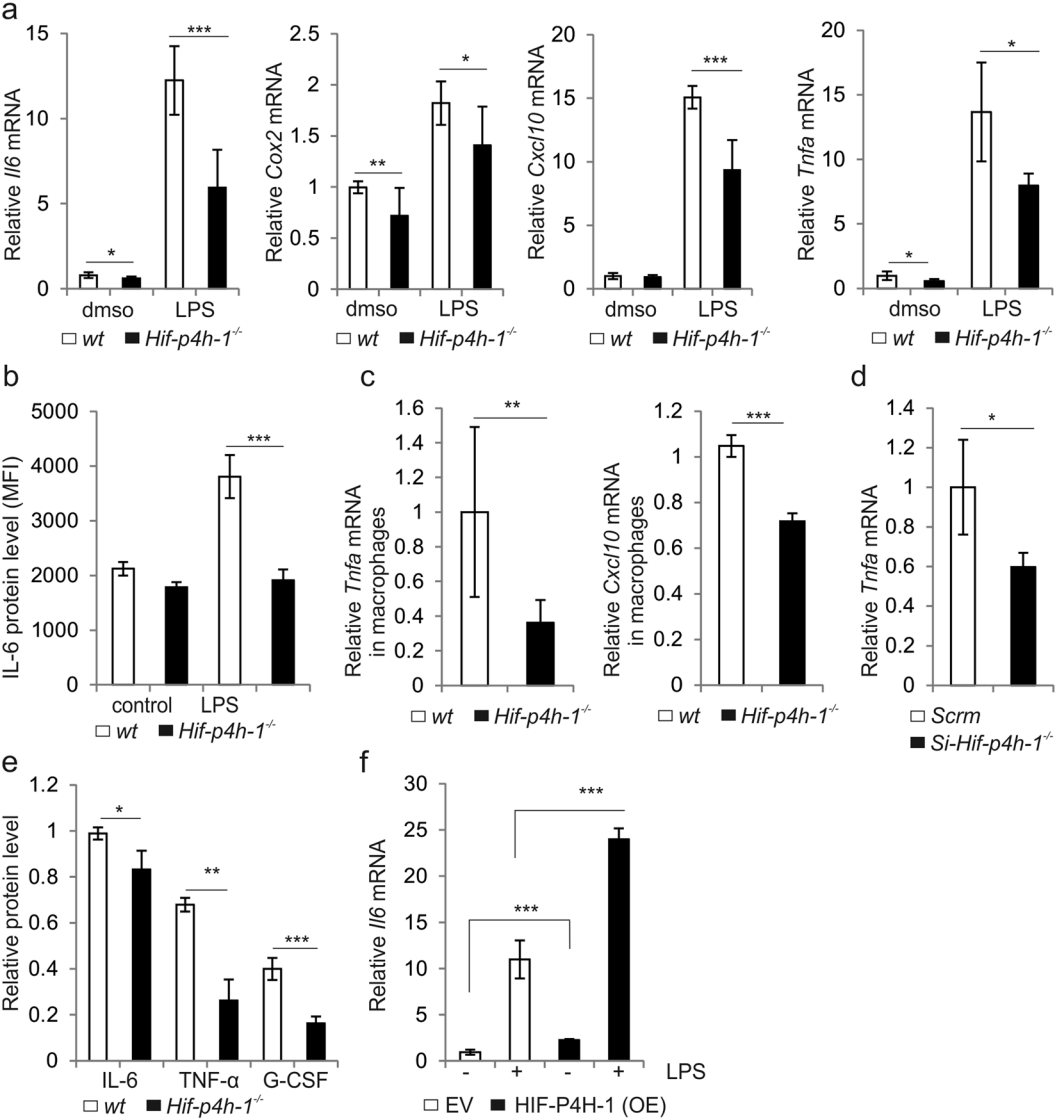
Lack of HIF-P4H-1 suppresses the expression of inflammatory genes under LPS stimulus. (**a**) qPCR analysis of the expression of genes for interleukin 6 (*Il6*), cyclooxygenase 2 (*Cox2*), *Cxcl10* and tumor necrosis factor α (*Tnfa*) in *Hif-p4h-1* ^−/−^ and wt MEFs treated with or without 200 ng/ml LPS for 12 h before extraction of total RNA. (**b**) FACS analysis of intracellular staining of IL-6 protein in wt and *Hif-p4h-1* ^−/−^ MEFs treated with or without 200 ng/ml LPS for 24 h. (**c**) qPCR analysis of the mRNA levels for TNF-α and CXCL10 in wt and *Hif-p4h-1* ^−/−^ macrophages treated with 10 ng/ml LPS for 72 h. (**d**) qPCR analysis of the mRNA level for TNF-α in *Hif-p4h-1* and scrambled (Scrm) siRNA transfected HEK293 cells. (**e**) ELISA of IL-6, TNF-α and G-CSF in culture medium of *Hif-p4h-1* ^−/−^ and wt MEFs treated with 200 ng/ml of LPS for 24h. (**f**) qPCR analysis of the mRNA level for IL-6 in *Hif-p4h-1* ^−/−^ MEFs transfected with empty vector (EV) or a vector encoding V5-tagged human recombinant HIF-P4H-1 (OE) and treated with or without 200 ng/ml LPS for 12 h. Data are presented as mean ± s.d., n = at least 3 individual wt and *Hif-p4h-1* ^−/−^ MEF or macrophage isolates (**a–c**) or individual siRNA and overexpression experiments (**d–f**) analysed in triplicates (**a**,**c–f**). *P < 0.05, **P < 0.01 and ***P < 0.001, two-tailed Student’s *t*-test.

To examine whether lack of HIF-P4H-1 affects gene expression under an inflammatory stimulus, the mRNA levels of selected proinflammatory cytokines and enzymes were analysed in LPS-induced (200 ng/ml, 12 h) *Hif-p4h-1* ^−/−^ and wt MEFs. The *Il6*, *Cox*2, *Cxcl10* and *Tnfa* mRNA levels were reduced in LPS-treated *Hif-p4h-1* ^−/−^ MEFs relative to wt (Fig. 2a). Selected further analyses confirmed these effects. Intracellular IL-6 staining was reduced in LPS-induced *Hif-p4h-1* ^−/−^ MEFs relative to wt (Fig. 2b). In addition to MEFs, the *Tnfa* and *Cxcl10* mRNA levels were reduced in LPS-induced (10 ng/ml, 72 h) *Hif-p4h-1* ^−/−^ macrophages relative to wt (Fig. 2c). Furthermore, *HIF-P4H-1* silencing by siRNA reduced the *Tnfa* mRNA in HEK293 cells (Fig. 2d). Consistent with the mRNA levels, the amounts of secreted IL-6, TNF-α and granulocyte-colony stimulating factor (G-CSF) protein were reduced in LPS-induced *Hif-p4h-1* ^−/−^ MEFs relative to wt (Fig. 2e). In contrast, overexpression of human HIF-P4H-1 in non-treated and LPS-induced *Hif-p4h-1* ^−/−^ MEFs resulted in higher *Il6* mRNA level (Fig. 2f). Thus, HIF-P4H-1 depletion reduces inflammatory gene expression in several cell types at baseline and under LPS stimulus.

### Lack of HIF-P4H-1 downregulates basal and LPS-induced NF-κB signaling

**Figure 3:**
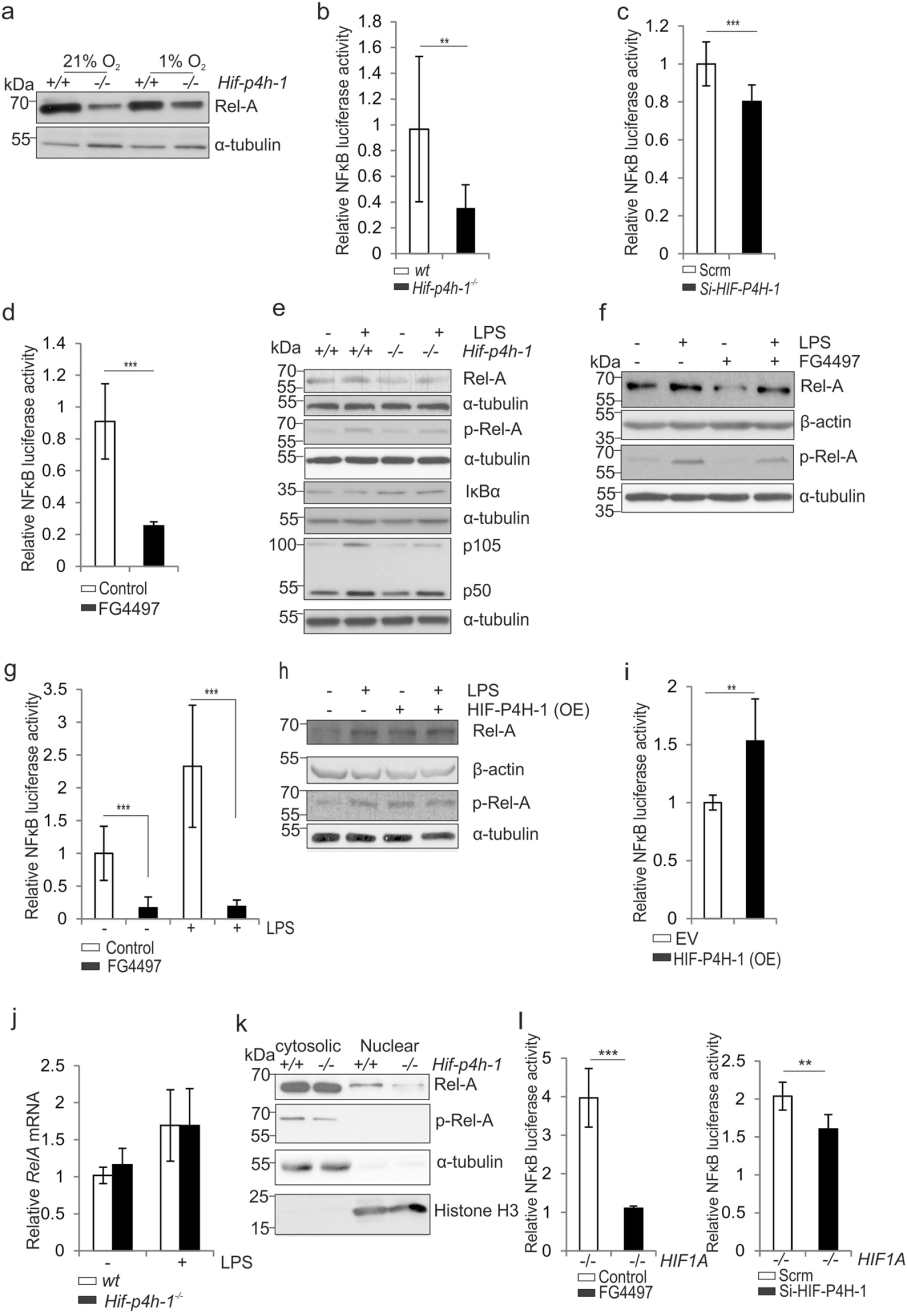
Lack of HIF-P4H-1 suppresses NF-κB activity. (**a**) Western blot analysis of Rel-A in wt and *Hif-p4h-1* ^−/−^ MEFs subjected to 21% or 1% O_2_ for 24 h. (**b**–**d**) Analysis of NF-κB luciferase reporter activity in wt and *Hif-p4h-1* ^−/−^ MEFs (**b**), in *Hif-p4h-1* and scrambled (Scrm) siRNA transfected HEK293 cells (**c**) and in MDA-MB-231 cells treated with 50 µM FG4497 for 6 h (**d**). Cells were harvested 48 h after the NF-κB luciferase reporter plasmid transfection. siRNA transfection was performed 24 h before the NF-κB luciferase reporter plasmid transfection. FG4497 was added 6 h before the cell harvest. (**e**) Western blot analysis of Rel-A, p-Rel-A, IκBα, p105 and p50 in wt and *Hif-p4h-1* ^−/−^ MEFs treated with or without 200 ng/ml LPS for 12 h. (**f,g**) Analysis of Rel-A and p-Rel-A by Western blotting (**f**) and NF-κB luciferase reporter activity (**g**) in wt MEFs treated with or without 200 ng/ml LPS and 50 µM FG4497 for 6 h. (**h**,**i**) Analysis of Rel-A and p-Rel-A by Western blotting (**h**) and NF-κB luciferase reporter activity (**i**) in *Hif-p4h-1* ^−/−^ MEFs transfected with empty vector (EV) or a vector encoding V5-tagged human recombinant HIF-P4H-1 (OE) and treated with or without 200 ng/ml LPS for 12 h. (**j**) qPCR analysis of Rel-A mRNA in *Hif-p4h-1* ^−/−^ and wt MEFs treated with 200 ng/ml of LPS for 12 h. (**k**) Western blot analysis of Rel-A and p-Rel-A in cytosolic and nuclear fractions of *Hif-p4h-1* ^−/−^ and wt MEFs. (**l)** NF-κB luciferase reporter activity in *HIF1A* ^−/−^ HCT116 cells treated with FG4497 or transfected with HIF-P4H-1 siRNA. Data are presented as representative Western blots and as mean ± s.d., n = 3–4 individual MEF isolates or experiments. *P < 0.05, **P < 0.01 and ***P < 0.001, two-tailed Student’s *t*-test. Unprocessed original scans of blots are shown in Supplementary Fig. 5.

Based on the above data (Fig. 1a,b, Fig. 2) and the proposed role of HIF-P4H-1 in regulation of NF-κB signaling^16,18^, we analysed the NF-κB complex proteins Rel-A/p65, p150 and p50^14,36^ in *Hif-p4h-1* ^−/−^ MEFs. The baseline Rel-A amount was lower in *Hif-p4h-1* ^−/−^ MEFs relative to wt in normoxia and hypoxia (Fig. 3a). Consistently, NF-κB reporter activity was reduced in *Hif-p4h-1* ^−/−^ MEFs relative to wt (Fig. 3b). A similar effect was obtained with siRNA silencing of *HIF-P4H-1* in HEK293 cells and with a pan HIF-P4H inhibitor FG4497^37^ (50 μM, 6 h) treatment of MDA-MB-231 cells (Fig. 3c,d). The amounts of Rel-A, p105 and p50 were lower also in LPS-induced (200 ng/ml, 12 h) *Hif-p4h-1* ^−/−^ MEFs relative to wt (Fig. 3e). Likewise, the Rel-A amount and NF-κB reporter activity were decreased in cells treated with FG4497 relative to controls at baseline and under LPS stimulus (Fig. 3f,g). In contrast, reconstitution of *Hif-p4h-1* ^−/−^ MEFs with human HIF-P4H-1 increased the Rel-A amount and NF-κB activity when compared to controls (Fig. 3h,i). To study what is the mechanism of the HIF-P4H-1-dependent decrease of Rel-A amount we first show by qPCR analysis that the reduction is not caused by decreased transcription of Rel-A (Fig. 3j). Regulation of Rel-A activity takes place by prevention of the phosphorylation and nuclear translocation of Rel-A through binding to an inhibitory IκB protein^10,14,15^. We observed a clear reduction in the phosphorylation and nuclear localization of Rel-A upon genetic or chemical HIF-P4H-1 inhibition (Fig. 3e,f and k), while in HIF-P4H-1 overexpressing cells the phosphorylation was increased relative to wt (Fig. 3h). We next studied whether the reduction of NF-κB activity upon inactivation of HIF-P4H-1 involves HIF1α and treated *HIF1A* ^−/−^ HCT-116 cells with *HIF-P4H-1* siRNA or FG4497. Both treatments resulted in decreased NF-κB luciferase activity in the *HIF1A* ^−/−^ cells (Fig. 3l) indicating that the effect of HIF-P4H inhibition is not dependent on HIF1α and that HIF-P4H-1 has direct, yet unidentified, targets in the NF-κB signaling pathway.

### Lack of HIF-P4H-1 increases cleaved caspase 3 and p53 amounts and cell death under apoptotic stimulus

**Figure 4:**
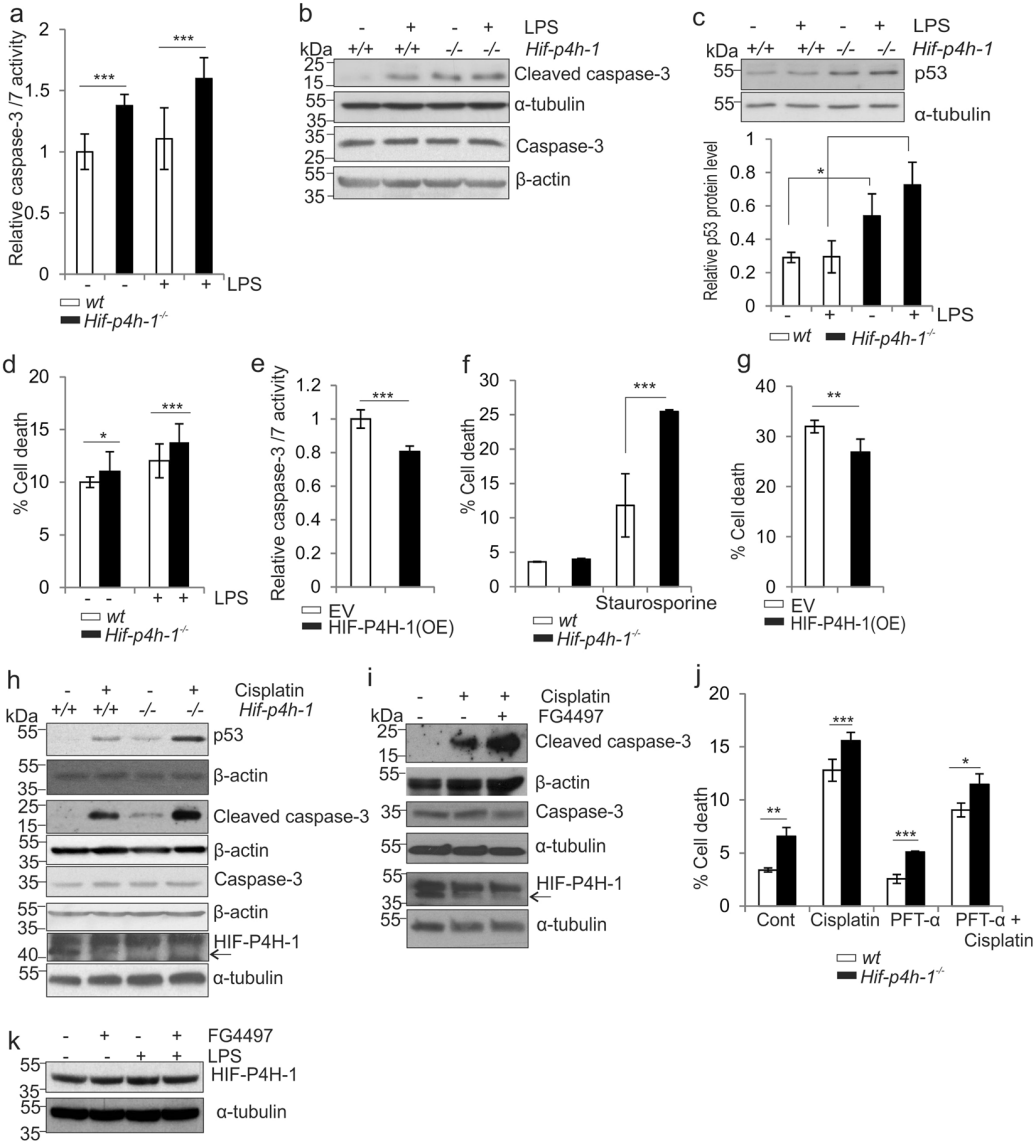
Lack of HIF-P4H-1 increases cell death. (**a**–**c**) Analysis of relative caspase 3/7 activity (**a**), caspase-3 and cleaved caspase-3 (**b**), and p53 (**c**) protein amounts in wt and *Hif-p4h-1* ^−/−^ MEFs treated with or without 100 ng/ml LPS for 24 h. (**d**) Analysis of viability of wt and *Hif-p4h-1* ^−/−^ MEFs treated with or without 5 mg/ml of LPS for 48h. Quantitation of cell viability based on 7-actinomycin D staining and FACS analysis is shown. (**e**) Analysis of relative caspase 3/7 activity in *Hif-p4h-1* ^−/−^ MEFs transfected with empty vector (EV) or a vector encoding V5-tagged human recombinant HIF-P4H-1 (OE). (**f**) Analysis of viability of wt and *Hif-p4h-1* ^−/−^ MEFs treated with or without 2 μM staurosporine for 24 h. (**g**) Analysis of viability of *Hif-p4h-1* ^−/−^ MEFs transfected with empty vector (EV) or a vector overexpressing (OE) V5-tagged human recombinant HIF-P4H-1 and treated with 2 μM staurosporine for 24 h. Quantitation of cell viability based on 7-actinomycin D staining and FACS analysis is shown. (**h**) Western blot analysis of p53, caspase-3, cleaved caspase-3 and HIF-P4H-1 in wt and *Hif-p4h-1* ^−/−^ MEFs treated with or without 10 μg/ml cisplatin for 6 h. (**i**) Western blot analysis of caspase-3, cleaved caspase-3 and HIF-P4H-1 in wt MEFs treated with or without 10 μg/ml cisplatin and 50 µM FG4497 for 6 h. (**j**) Analysis of viability of *Hif-p4h-1* ^−/−^ and wt MEFs treated with or without 50 μg/ml cisplatin and 100 μM PFT-α for 24 h. Quantitation of cell viability based on dynamic imaging of live and dead cells in the IncuCyte^TM^ zoom live-cell imaging system is shown. (**k**) Western blot analysis of HIF-P4H-1 in wt HCT-116 cells treated with or without 100 ng/ml LPS and 50 µM FG4497 for 6 h. Data are presented as representative Western blots and as mean ± s.d., n = 3 individual MEF isolates or experiments. *P < 0.05, **P < 0.01 and ***P < 0.001, two-tailed Student’s *t*-test. Unprocessed original scans of blots are shown in Supplementary Fig. 5.

As the gene expression data showed that *Perp* and *Bnip3* mRNA levels were increased in *Hif-p4h-1* ^−/−^ MEFs relative to wt (Fig. 1c, supplementary Table 2), we analysed apoptosis of these cells. Activity of the proapoptotic effectors caspase 3 and 7, the amounts of cleaved caspase 3 and p53, and cell death were higher in *Hif-p4h-1* ^−/−^ MEFs relative to wt at baseline and under LPS stimulus (200 ng/ml, 12 h) (Fig. 4a–d). In contrast, overexpression of human HIF-P4H-1 in *Hif-p4h-1* ^−/−^ MEFs reduced caspase-3/7 activity (Fig. 4e). We next treated the cells with staurosporine (2 μM, 24 h) that induces both caspase dependent and independent apoptosis^38,39^. The *Hif-p4h-1* ^−/−^ MEFs were more sensitive to staurosporine-induced cell death (Fig. 4f), while overexpression of human HIF-P4H-1 in *Hif-p4h-1* ^−/−^ MEFs partially reversed this effect (Fig. 4g). The chemotherapeutic agent cisplatin increases p53 accumulation and apoptosis^40^. Cisplatin treatment also resulted in higher amounts of p53, cleaved caspase-3, and cell death in *Hif-p4h-1* ^−/−^ MEFs relative to wt (Fig. 4h–j). Treatment with 50 μM FG4497 mimicked genetic HIF-P4H-1 inhibition in that cleaved caspase 3 amount was increased under cisplatin treatment (Fig. 4i). We also analyzed whether any of the treatments affected HIF-P4H-1 protein level. LPS and FG4497 had no effect (Fig. 4k), while cisplatin led to a systematic decrease in HIF-P4H-1 protein amount (Fig. 4h,i). It may be noted that although HIF-P4H-1 knockout alone consistently resulted in increased cleaved caspase-3 amount and caspase-3/7 activity (Fig. 4a,b and h), this difference between the genotypes did not always manifest at the level of actual cell death at baseline conditions, but was consistently observed when the cells were challenged with LPS, staurosporine or cisplatin (Fig. 4d,f and j). To further study whether the observed higher amount of p53 is responsible for increased cell death of *Hif-p4h-1* ^−/−^ MEFs under an apoptotic stimulus, we treated the cells with pifithrin-α (PFT-α), a p53 inhibitor, in the presence or absence of cisplatin. As expected, PFT-α treatment reduced cell death, especially under the cisplatin treatment, but notably the difference between the genotypes persisted reflecting the difference in the p53 amount (Fig. 4j).

### Lack of HIF-P4H-1 increases p53 protein half-life

**Figure 5:**
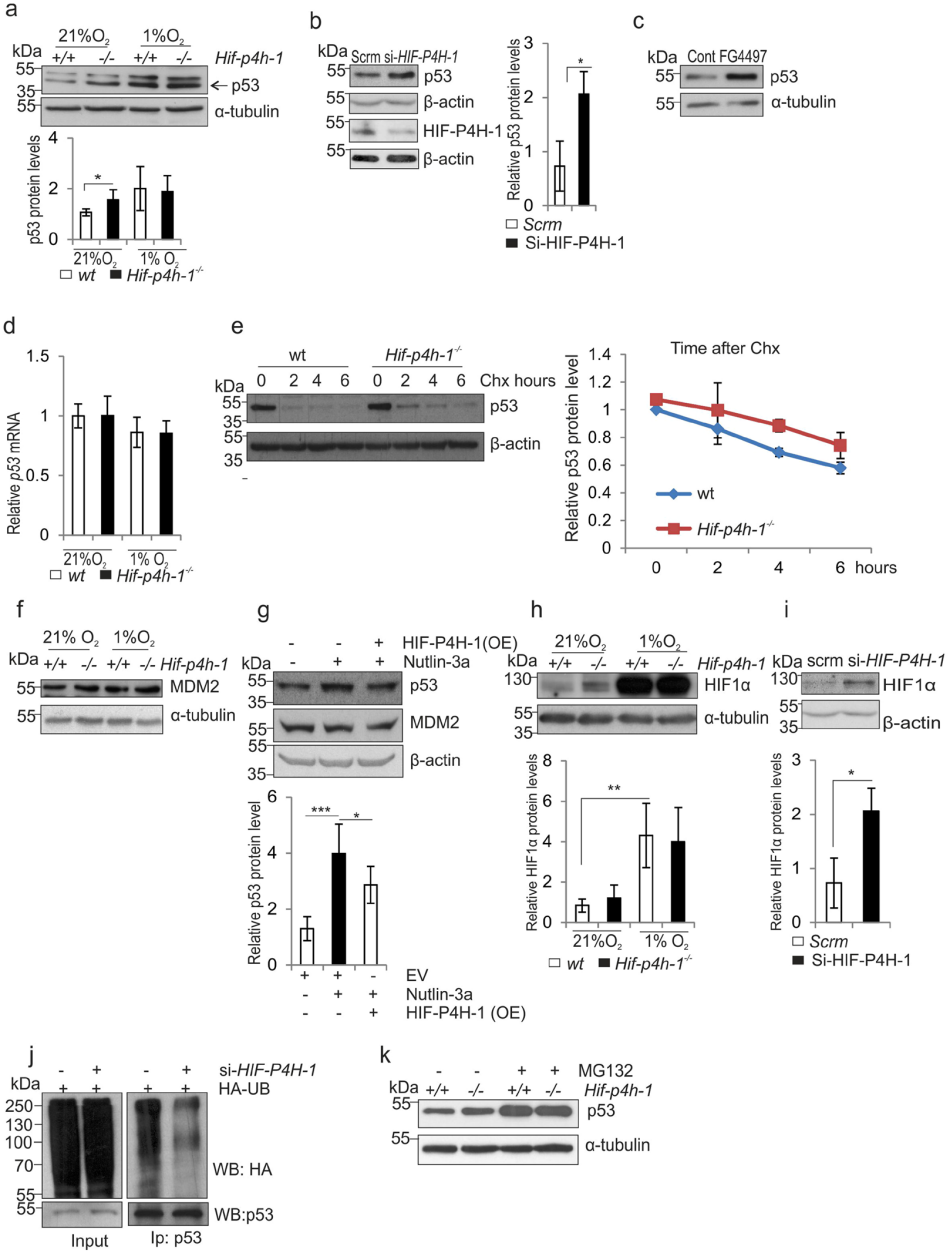
Lack of HIF-P4H-1 increases the amount of p53. (**a**) Western blot analysis of p53 in wt and *Hif-p4h-1* ^−/−^ MEFs cultured in 21% or 1% O_2_ for 24 h. (**b**,**c**) Western blot analysis of p53 in *Hif-p4h-1* and scrambled (Scrm) siRNA transfected HEK293 cells (**b**) and wt MEFs treated with 50 µM FG4497 for 24 h (**c**). (**d**) qPCR analysis of p53 mRNA in wt and *Hif-p4h-1* ^−/−^ MEFs cultured in 21% or 1% O_2_ for 24 h. (**e)** Analysis of p53 protein turnover rate. *Hif-p4h-1* ^−/−^ and wt MEFs were treated with 200 μg/ml of cycloheximide for the indicated time points and whole cell lysates were blotted for p53. (**f**) Western blot analysis of MDM2 in wt and *Hif-p4h-1* ^−/−^ MEFs cultured in 21% or 1% O_2_ for 24 h. (**g**) Western blot analysis of p53 and MDM2 in wt MEFs treated with 10 μM nutlin-3a for 24 h with or without overexpression of human HIF-P4H-1 (OE). (**h**,**i**) Western blot analysis of HIF1α in wt and *Hif-p4h-1* ^−/−^ MEFs cultured in 21% or 1% O_2_ for 24 h (**h**) and in scrambled and *Hif-p4h-1* siRNA transfected HEK293 cells (**i**). (**j**) Western blot analysis of ubiquitination of p53 in *Hif-p4h-1* and scrambled siRNA transfected HEK293 cells. The cells were transfected with HA-ubiquitin and endogenous p53 was immunoprecipitated followed by Western blotting with anti-HA and anti-P53 antibodies. (**k**) Western blot analysis of p53 in *Hif-p4h-1* ^−/−^ and wt MEFs were treated with or without 10 μM MG132 for 5 h. Data are presented as representative Western blots and as mean ± s.d., n = at least 3 individual MEF isolates or experiments. *P < 0.05, **P < 0.01 and ***P < 0.001, two-tailed Student’s *t*-test. Unprocessed original scans of blots are shown in Supplementary Fig. 5.

Hypoxia and HIF1α influence the stability and activity of p53 in diverse ways and in cell line dependent manner^27^. To study the link between HIF-P4H-1 and p53 protein amount, we cultured *Hif-p4h-1* ^−/−^ and wt MEFs in normoxia and hypoxia. As above (Fig. 4c,h), p53 amount was higher in *Hif-p4h-1* ^−/−^ MEFs than in wt in normoxia, but accumulated to a similar extent in both genotypes in hypoxia (Fig. 5a). Similarly, siRNA silencing of *HIF-P4H-1* in HEK293 cells (Fig. 5b) and FG4497 treatment of wt MEFs (Fig. 5c) increased p53 amount. The higher p53 protein amount in *Hif-p4h-1* ^−/−^ MEFs in normoxia was not caused by increased transcription (Fig. 5d). Furthermore, analysis of p53 protein degradation following inhibition of protein synthesis by cycloheximide treatment showed that the half-life of p53 was increased in *Hif-p4h-1* ^−/−^ MEFs (Fig. 5e). These data suggest that the HIF-P4H-1 mediated effect on p53 protein occurs at the post-transcriptional level.

The p53 protein is normally maintained at low levels by MDM2^41,42^. It has been reported that hypoxia downregulates MDM2 protein level, MDM2 mediates an indirect interaction between HIF1α and p53, and that HIF1α protects p53 from degradation^43–45^, but direct interaction between HIF1α and p53 has also been shown^46,47^. We observed no marked differences in MDM2 protein level in *Hif-p4h-1* ^−/−^ and wt MEFs in normoxia and hypoxia (Fig. 5f). Furthermore, increased p53 amount in wt MEFs generated by MDM2 antagonist nutlin-3a (10 μM, 24 h), was stifled by simultaneous overexpression of human HIF-P4H-1 without affecting the MDM2 level (Fig. 5g). The HIF1α amount was increased in normoxia in *Hif-p4h-1* ^−/−^ MEFs (Fig. 5h) and in *HIF-P4H-1* siRNA transfected HEK293 cells relative to the controls (Fig. 5i). These data suggest that the HIF-P4H-1 mediated effect on p53 protein does not involve MDM2, but HIF1α may have a role.

Hydroxylation of HIF1α by HIF-P4Hs leads to its ubiquitination and proteasomal degradation and lack of HIF-P4H function inhibits these subsequent events. We next studied whether inactivation of HIF-P4H-1 has similar effects on p53. siRNA silencing of HIF-P4H-1 reduced ubiquitylation of p53 in HEK293 cells (Fig. 5j). Furthermore, equal accumulation of p53 protein was observed in *Hif-p4h-1* ^−/−^ MEFs relative to wt upon inhibition of proteasomal degradation (Fig. 5k). These data indicate that the effects of HIF-P4H-1 on p53 are analogous to those on HIF1α.

### HIF-P4H-1 interacts with p53 and hydroxylates it at Pro142

**Figure 6:**
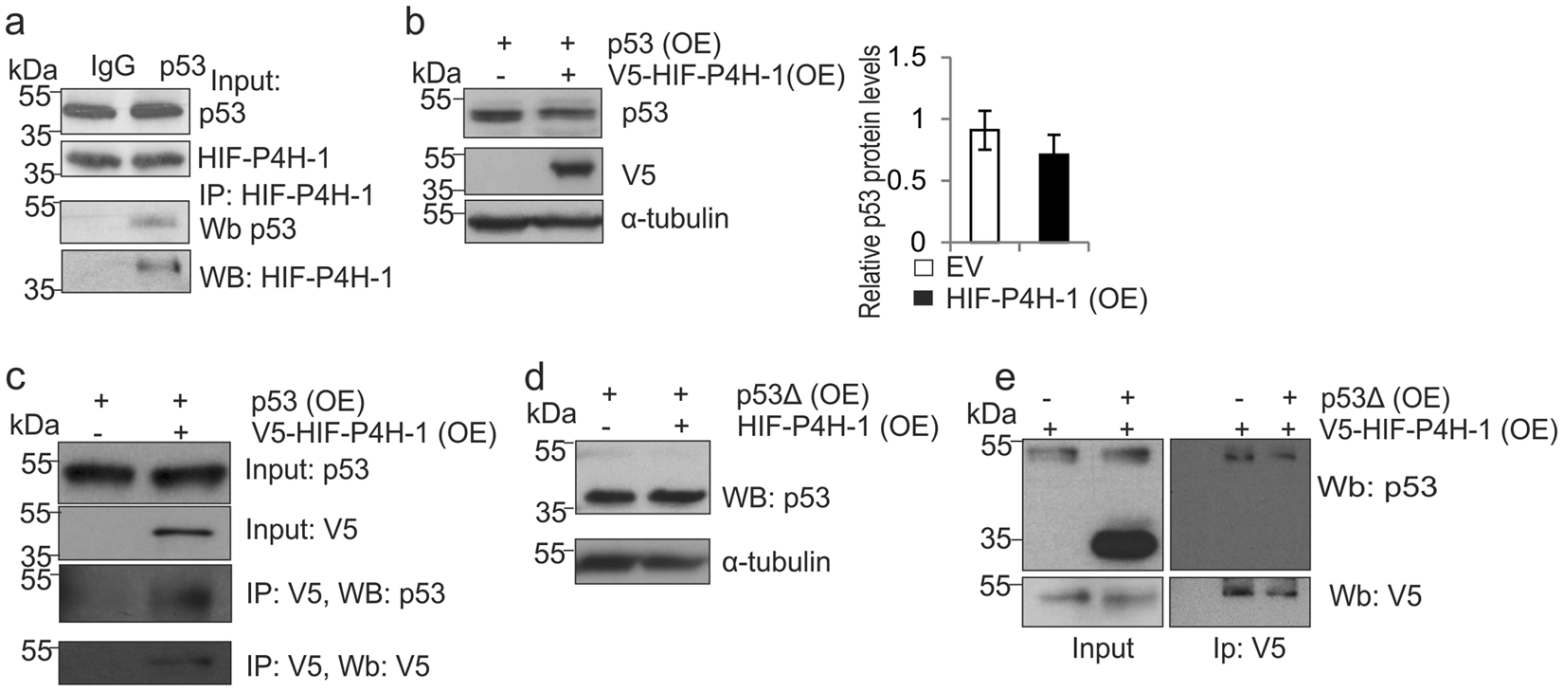
HIF-P4H-1 interacts with p53. (**a**) Immunoprecipitation of endogenous p53 from wt MEFs treated with 10 μM MG132 for 4 h before cell lysis. Co-immunoprecipitation of endogenous HIF-P4H-1 was analyzed by Western blotting with an anti-HIF-P4H-1 antibody. (**b**) HEK293 cells were transfected with a plasmid encoding full-length p53 together with a plasmid encoding V5-tagged human HIF-P4H-1 (OE) or an empty vector (EV) and analysed by Western blotting with anti-p53 and anti-V5 antibodies. (**c**) HEK293 cells were transfected as in (**b**) treated with 10 μM MG132 for 4 h and immunoprecipitated with an anti-V5 antibody. Co-immunoprecipitation of p53 was analysed by Western blotting with an anti-p53 antibody. (**d**) HEK293 cells were transfected with a plasmid encoding a deleted form of p53 that lacks amino acid residues 75–207 (p53Δ) together with a plasmid encoding V5-tagged human HIF-P4H-1 (OE) or an empty vector and analysed by Western blotting with anti-p53 and anti-V5 antibodies. (**e**) HEK293 cells were transfected as in **d**, treated with 10 μM MG132 for 4 h and immunoprecipitated with an anti-V5 antibody. Co-immunoprecipitation of p53 was analysed by Western blotting with an anti-p53 antibody. Data are presented as representative Western blots and as mean ± s.d., n = at least 3 individual experiments. Unprocessed original scans of blots are shown in Supplementary Fig. 5.

Based on the above data we set out to study whether HIF-P4H-1 interacts with p53 and performed coimmunoprecipitation from wt MEFs treated with the proteasomal inhibitor MG132 (10 μM, 4 h) with anti-p53 antibody followed by Western blotting with anti-HIF-P4H-1 antibody, and detected HIF-P4H-1 in the immunoprecipitates (Fig. 6a). To study this further we overexpressed p53 with and without V5-tagged HIF-P4H-1 in HEK293 cells. Consistent with the above data, overexpression of V5-HIF-P4H-1 reduced p53 amount (Fig. 6b) and p53 coimmunoprecipitated with V5-HIF-P4H-1 (Fig. 6c).

HIF-P4Hs hydroxylate two prolines in HIF1α in a -Leu-X-X-Leu-Ala-Pro- sequence^3–7,48^. However, HIF-P4Hs hydroxylate synthetic peptides with various substitutions in the -Leu-X-X-Leu- sequence^49^ and subsequently several other proteins have been implicated to be hydroxylated by HIF-P4Hs in -Leu-X-X-Leu-Ala-Pro- and other sequences^8,18^. p53 contains several prolines, including one, Pro190, in a -Leu-Ala-Pro- context. Therefore, we studied HIF-P4H-1 interaction with a deleted form of p53 lacking Pro190, p53Δ75-207. In contrast to decreasing full-length p53 amount (Fig. 6b), V5-HIF-P4H-1 overexpression did not affect p53Δ75-207 amount (Fig. 6d), suggesting that HIF-P4H-1 may not interact with p53Δ75-207. Endogenous full-length p53, but not p53Δ75-207, was coimmunoprecipitated with V5-HIF-P4H-1 (Fig. 6e), indicating that the deleted region is required for the interaction or, alternatively, the deletion hinders p53 folding in which case the interaction with HIF-P4H-1 would require correct p53 conformation.

**Figure 7:**
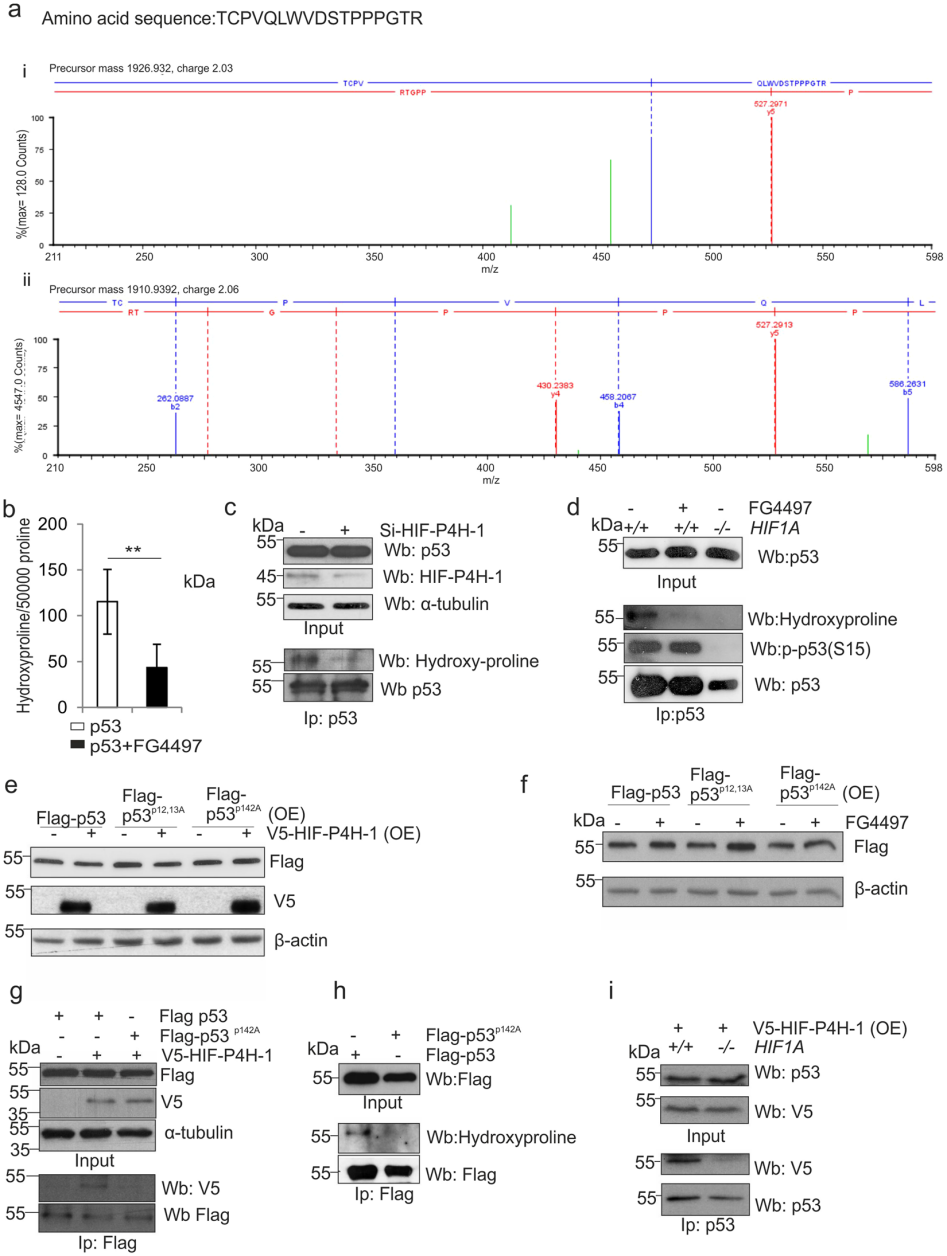
p53 is hydroxylated at Pro142. (**a**) Flag-tagged p53 was immunoprecipitated from HCT-116 cells treated with 10 μg/ml cisplatin and 10 μM MG132 for 6 h, separated by SDS-PAGE, trypsinized and analyzed by LC-MS. Mass signals extracted from the high energy trace of the MS^e^ measurement assigned to the peptide TCPVQLWVDSTPPPGTR (140–156) are shown. Low molecular weight area for the oxidized (i) and unmodified (ii) peptide with the b4 ions was used to assign hydroxylation to Pro142. Detailed information on all found fragment ions are provided in Supplementary tables 5 and 6. (**b**) [2,3,4,5-^3^H] proline-labeled Flag-tagged p53 was produced in wt HCT-116 cells treated with 10 μg/ml cisplatin and 10 μM MG132 with or without 50 μM FG4497 for 6 h. Flag-p53 was immunoprecipitated and the amount of 4-hydroxy[^3^H]proline formed was measured by a radiochemical method. The data are given as the amount of 4-hydroxyproline residues/50 000 proline residues. (**c**) HCT-116 cells were transfected with scrambled *or HIF-P4H-1* siRNA and treated with 10 μg/ml cisplatin and 10 μM MG132 for 6 h. Endogenous p53 was immunoprecipitated and analysed by Western blotting with antibodies agains hydroxyproline and p53. (**d**) Wt and *HIF1A* ^−/−^ HCT-116 cells were treated with 10 μg/ml cisplatin and 10 μM MG132 for 6 h with or without 50 μM FG4497. Endogenous p53 was immunoprecipitated and analysed by Western blotting with antibodies against hydroxyproline, P(Ser15)-p53 and p53. (**e**,**f**) Flag-tagged wt, Pro12Ala/Pro13Ala and Pro142Ala p53 were overexpressed (OE) in HCT-116 cells with or without V5-tagged HIF-P4H-1 (**e**) and with or without 50 μM FG4497 for 6 h (**f**) and the amount of p53 and HIF-P4H-1 was analysed by Western blotting with anti-Flag and anti-V5 antibodies, respectively. (**g**) Flag-tagged wt and Pro142Ala p53 and V5-tagged HIF-P4H-1 were overexpressed in HEK293 cells and treated with 10 μM MG132 for 6 h. Flag-tagged p53 proteins were immunoprecipitated and co-immunoprecipitation of HIF-P4H-1 was analysed by Western blotting with an anti-V5 antibody. (**h**) Flag-tagged wt and Pro142Ala p53 were overexpressed in HCT-116 cells and treated with 10 μg/ml cisplatin and 10 μM MG132 for 6 h. Flag-p53 was immunoprecipitated and hydroxylation was analysed by Western blotting with an anti-hydroxyproline antibody. (**i**) V5-tagged HIF-P4H-1 was overexpressed (OE) in wt and *HIF1A* ^−/−^ HCT-116 cells treated with 10 μM MG132 for 6 h. Endogenous p53 was immunoprecipitated and co-immunoprecipitation of HIF-P4H-1 was analysed by Western blotting with an anti-V5 antibody. Data are presented as representative Western blots and as mean ± s.d., n = at least 3 individual experiments in (**b**–**i**). *P < 0.05, **P < 0.01 and ***P < 0.001, two-tailed Student’s *t*-test. Unprocessed original scans of blots are shown in Supplementary Fig. 5.

We next asked whether interaction between HIF-P4H-1 and p53 results in hydroxylation of p53 prolyl residue(s). We immunoprecipitated overexpressed Flag-tagged p53 from HCT-116 cells treated with cisplatin (10 μg/ml) and MG132 (10 μM) for 6 h and subjected it to LC-MS analysis. We obtained ~70% p53 sequence coverage and evidence for substantial post-translational modification, such as phosphorylation, N-terminal acetylation, deamidation and oxidations. There were several oxidized p53 peptides containing proline(s), which could indicate proline hydroxylation, but the MS/MS data did not allow unambiguous identification of the oxidized residue, and in most cases it could have been methionine oxidation, a common artefact. However, a particular p53 peptide with no methionine (TCPVQLWVDSTPPPGTR, residues 140–156), appeared in both oxidized and non-oxidized form (Fig. 7a). Although the highest score to explain the oxidation (P or W) was obtained for hydroxylation of Pro142, the score difference is marginal and does not allow reliable mapping of the oxidation site (supplementary Table 7). However, comparison of the MS/MS spectra between oxidized and non-oxidized peptide reveals discriminating b4 ions, which suggest hydroxylation at Pro142 (Fig. 7a, supplementary Fig. 3a). The ratio of the hydroxylated peptide was calculated to be 3–5%. We repeated the experiment in parallel with wt and Pro142Ala p53, and the Pro142Ala p53 did not show any evidence for oxidation in the peptide 142–156 (supplementary Fig. 3b). Because of the limitations of the MS/MS data we carried out several additional independent approaches to study further the potential hydroxylation of p53 and whether Pro142 is a hydroxylation target.

We tested whether synthetic p53 peptides TCP^142^VQLWVDSTPPPGTR and RCSDSDLAP^190^PQHLIRVEG are hydroxylated by recombinant human HIF-P4H-1 *in vitro* ^48,50,51^. Unlike with the HIF1α peptide DLDLEMLAPYIPMDDDFQL, no HIF-P4H-1 activity was detected with the two p53 peptides (data not shown). As short peptides may not be sufficient for interaction with and subsequent hydroxylation by HIF-P4H-1, we also produced full-length p53 in an *in vitro* transcription-translation system in the presence of L-[2,3,4,5-^3^H] proline, subjected it to hydroxylation by recombinant human HIF-P4H-1 and measured the amount of 4-hydroxy[^3^H]proline formed^50^, but in contrast to full-length HIF1α, obtained no evidence for p53 hydroxylation (data not shown).

The above data prompted us to study whether the potential hydroxylation of p53 Pro142 only occurs in a cellular context. We overexpressed Flag-tagged p53 in HCT-116 cells in the presence of L-[2,3,4,5-^3^H] proline and treated the cells with 10 μg/ml cisplatin, 10 μM MG132, with or without 50 μM FG4497 for 6 h. On average 90 4-hydroxyproline residues were detected per 50 000 proline residues in immunoprecipitated p53 in the absence of FG4497, i.e. 0.18% of total proline, while FG4497 reduced this by about 35% (Fig. 7b). Assuming that only a single proline is the hydroxylation target in p53, the maximum 4-hydroxyproline/proline ratio can be 2.2% (1/45 proline residues), and the obtained 0.18% ratio indicates that 8% of p53 produced under these experimental conditions became hydroxylated. Furthermore, Western blotting with an anti-hydroxyproline antibody showed that p53 is hydroxylated in HCT-116 cells and that siRNA silencing of HIF-P4H-1 or treatment with FG4497 resulted in a concomitant reduction in p53 hydroxylation (Fig. 7c,d).

As Pro142 was identified as a potential hydroxylation target we coexpressed wt and Pro142Ala p53 with and without HIF-P4H-1 and studied the p53 protein amount. As a control we used a Pro12Ala/Pro13Ala p53, where the mutated prolines are outside the amino acid 75–207 region. Simultaneous overexpression of HIF-P4H-1 reduced wt and Pro12Ala/Pro13Ala p53 levels, but not Pro142Ala p53 level (Fig. 7e). In contrast, wt and Pro12Ala/Pro13Ala p53 levels were increased by FG-4497, while Pro142Ala p53 level was not affected (Fig. 7f). Furthermore, immunoprecipitation showed that the p53 Pro142Ala mutation abolished the interaction between p53 and HIF-P4H-1 (Fig. 7g). Also, Western blotting with an anti-hydroxyproline antibody indicated that wt p53 becomes hydroxylated, while Pro142Ala p53 does not (Fig. 7h).

We next studied whether the HIF-P4H-1 mediated effect on p53 protein level involves HIF1α. We overexpressed V5-HIF-P4H-1 in MG132-treated (10 μM, 6 h) wt and *HIF1A* ^−/−^ HCT-116 cells and immunoprecipitated p53. HIF-P4H-1 coimmunoprecipitated with p53 from wt but not *HIF1A* ^−/−^ cells (Fig. 7i). We then analysed hydroxylation of endogenous p53 in wt and *HIF1A* ^−/−^ HCT-116 cells treated with 10 μ μg/ml cisplatin and 10 μM MG132 with or without 50 μM FG4497 for 6 h by Western blotting. FG4497 abolished hydroxylation, but did not affect p53 phosphorylation in wt cells (Fig. 7d). In contrast, no p53 hydroxylation or phosphorylation was detected in *HIF1A* ^−/−^cells (Fig. 7d). These data suggest that HIF1α is required for the interaction between HIF-P4H-1 and p53 and subsequent p53 hydroxylation. The data also show that p53 hydroxylation is not required for p53 phosphorylation, but the presence of HIF1α is.

### Lack of HIF-P4H-1 reduces acute inflammatory response and increases apoptosis in TPA-treated mouse skin

**Figure 8:**
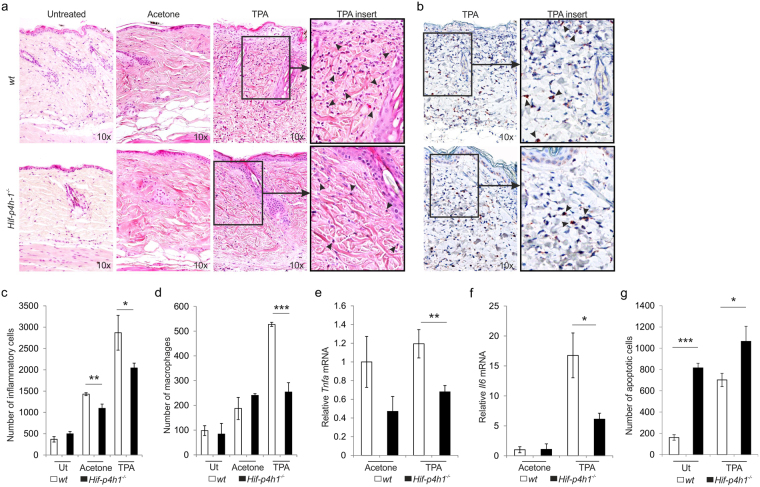
Lack of HIF-P4H-1 reduces the acute inflammatory response and increases apoptosis in mouse skin. The skin of wt and *Hif-p4h-1* ^−/−^ mice was topically treated with 12-O-tetradecanoylphorbol-13-acetate (TPA) and samples were collected after 24 h. (**a**) Histological analysis of the infiltration of inflammatory cells into the dermis (*arrows in inserts*) stained by hematoxylin and eosin. (**b**) Histological analysis of the infiltration of macrophages into the dermis (*arrows in inserts*) stained by Mac-3 immunostaining. (**c**) Morphometric analysis of inflammatory cell infiltration in untreated (Ut) mice was performed from 4–5 sections/mouse and 6 mice/genotype, in acetone-treated mice from 4–5 sections/mouse from 5 wt mice and 4 *Hif-p4h-1* ^−/−^ mice and in TPA-treated mice from 4–5 sections/mouse from 9 wt mice and 14 *Hif-p4h-1* ^−/−^ mice. (**d**) Morphometric analysis of macrophage infiltration in untreated mice was performed from 4–5 sections/mouse and 5 mice/genotype, in acetone-treated mice from 4–5 sections/mouse and 3 mice/genotype and in TPA-treated mice from 4–5 sections/mouse and 6 wt mice and 12 *Hif-p4h-1* ^−/−^ mice. The data are shown as mean number of cells/skin mm^2^ ± s.e.m. (**e**,**f**) qPCR analysis of the mRNA levels for TNF-α (**e**) and IL-6 (**f**) in the skin. Data are presented as mean ± s.e.m., n = 3 for acetone-treated mice, n = 14 for TPA-treated wt mice and n = 18 for TPA-treated *Hif-p4h-1* ^−/−^ mice analysed in duplicates. (**g**) Morphometric analysis of apoptotic cells in untreated (Ut) mice was performed from 4 sections/mouse and 4 wt mice and 3 *Hif-p4h-1* ^−/−^ mice and in TPA-treated mice from 4 sections/mouse and 11 mice/genotype. The data are shown as mean number of apoptotic cells/skin mm^2^ ± s.e.m. *P < 0.05, **P < 0.01 and ***P < 0.001, two-tailed Student’s *t*-test (untreated and acetone-treated samples in **c**, all samples in **f**), paired Student’s *t*-test (TPA-treated samples in **c**), and Mann-Whitney U test (**d**,**e**).

To evaluate potential *in vivo* relevance of the above findings, we subjected the skins of wt and *Hif-p4h-1* ^−/−^ mice to acute inflammation via topical application of 12-O-tetradecanoylphorbol-13-acetate (TPA)^52^. The number of intradermal inflammatory cells/macrophages was significantly lower in *Hif-p4h-1* ^−/−^ mice than in wt 24 h after TPA application (Fig. 8a–d). Vehicle treatment alone caused some irritation and according to H&E staining there were less infiltrating inflammatory cells in *Hif-p4h-1* ^−/−^ vehicle group than in wt, but this was not evident in the Mac-3 stained samples (Fig. 8a–d). qRT-PCR showed that *Tnfa* and *Il6* mRNA levels were significantly lower in TPA-treated *Hif-p4h-1* ^−/−^ skin than in wt (8e,f). In contrast, the number of cells undergoing apoptosis was significantly higher in both untreated and TPA-treated *Hif-p4h-1* ^−/−^ mouse skin than in wt (Fig. 8g, supplementary Fig. 4). These data indicate that acute inflammatory response is dampened and apoptotic activity is increased in *Hif-p4h-1* ^−/−^ skin relative to wt.

## Discussion

In normoxia HIF-P4Hs restrain the hypoxia response programme via targeting HIF1α for proteasomal degradation. Reduced oxygenation characterizes many pathological conditions HIF-P4H inhibitors are regarded as potential therapeutics to enhance the hypoxia response in such diseases^8,9,13,53,54^. Several companies are developing HIF-P4H inhibitors, the most advanced one being in phase 3 clinical trials to treat anemia of chronic kidney disease via inducing erythropoietin (EPO) production^54^. HIF-P4H-2 is the major HIF regulator and its inactivation alone induces EPO production robustly, although HIF-P4H-1 and 3, and a fourth putative HIF-P4H, a transmembrane P4H, also contribute to this^8,37,55–57^. In addition, HIF-P4Hs participate in the regulation of metabolism and damage control of ischemic insults^8,9,12,53,58–63^. HIF-P4H-1 and 3 have been implicated in the regulation of inflammatory pathways, and their chemical or genetic inhibition has beneficial effects in mouse models of inflammatory bowel disease^20–25^. Furthermore, HIF-P4H-1 has been reported to affect the regulation of inflammatory NF-κB signaling^16–19^.

We found that, in general, expression of proinflammatory genes was downregulated in *Hif-p4h-1* ^−/−^ MEFs and macrophages and in HIF-P4H-1 silenced HEK293 cells, while overexpression of HIF-P4H-1 had an opposite effect. These differences were prominent under LPS stimulus, but were already observed at baseline. The repressive effect of HIF-P4H-1 inhibition on proinflammatroy genes was supported by both basal and LPS-induced NF-κB activity and Rel-A protein amount and phosphorylation being reduced in cells where HIF-P4H-1 genetically inactivated or the cells were treated with a HIF-P4H inhibitor. This agrees with previous data showing that HIF-P4H-1 silencing or treatment with HIF-P4H inhibitors reduces LPS and IL-1β induced NF-κB activity in macrophages and HeLa cells, respectively^17,18^. The effect of HIF-P4H-1 inhibition on IL-1β-induced NF-κB activity has been suggested to involve hydroxylation of several proteins of the distal IL-1β signaling pathway, but this remains to be confirmed^18^. In contrast to our data, HIF-P4H-1 silencing by siRNA has been shown to induce basal NF-κB activity in HeLa and HEK293 cells, while overexpression of HIF-P4H-1 inhibited NF-κB activity in the latter^16,19^. In addition, elevated basal NF-κB activity has been observed in *Hif-p4h-1* ^−/−^ mouse hepatocytes resulting in NF-κB-dependent reduction of apopotosis^64^. The repressive effect of HIF-P4H-1 on basal NF-κB activity was suggested to be via hydroxylation and concomitant inactivation of IKKβ, which results in failure of IKKβ phosphorylating the inhibitor IκBα and liberation of NF-κB from the inactive NF-κB/ IκBα complex, inhibition of HIF-P4H-1 thus leading to induced basal NF-κB activity^16^. Despite these discrepancies regarding the role of HIF-P4H-1 in the regulation of basal NF-κB activity, it seems clear that HIF-P4H-1 inhibition has beneficial effects under an inflammatory stimulus, which is further supported by our results that *Hif-p4h-1* ^−/−^ mice show less inflammation in an acute skin inflammation model and by several reports on protective effects of genetic inactivation of HIF-P4H-1 or treatment with HIF-P4H inhibitors in inflammatory bowel disease models^20–26^.

Besides the hypoxia response and NF-κB pathways, the tumor suppressor p53 has important roles in the regulation of inflammation as it is regarded as a negative regulator of inflammation, and the intertwining of these pathways has been reviewed extensively^10,27,28,30–33,36^. We wanted to study whether HIF-P4H-1 has a role in the reciprocal antagonistic relationship between the p53 and NF-κB pathways. We found that inactivation or inhibition of HIF-P4H-1 led to accumulation of p53, increased expression of proapoptotic genes, increased caspase 3/7 activity and apoptosis in various cell types, both at basal conditions and under LPS stimulus or treatment with apoptosis inducing agents. We also showed that lack of HIF-P4H-1 has a proapoptotic effect in an acute *in vivo* skin inflammation model. These data suggest that inactivation of HIF-P4H-1 increases the activity of the p53 pathway, which in turn can contribute to the suppression of NF-κB activity. We demonstrate that HIF-P4H-1 physically interacts with p53 and that it hydroxylates at least p53 Pro142, this hydroxylation affecting p53 stability. A previous study has reported that silencing of HIF-P4H-1 prevents p53 activation upon chemotherapy in various colorectal cancer cell lines, which inhibits DNA repair and favors cell death^34^. Furthermore, by using dimethyloxaloylglycine (DMOG) they provided evidence that HIF-P4H-1 hydroxylase activity promotes p53 phosphorylation at Ser15 and this is not dependent on HIF1α. However, no direct evidence of p53 proline hydroxylation was presented. Our data show that HIF-P4H-1 inhibition increases the p53 accumulation, p53 Pro142 hydroxylation does not occur in cells that lack HIF1α, and furthermore, the p53 Pro142 hydroxylation status does not affect p53 Ser15 phosphorylation, but the latter requires the presence of HIF1α. Despite the potential differences in the observed mechanisms, the observed net effect in our study and that reported by Deschoemaeker and coworkers^34^ is the same, HIF-P4H-1 inactivation leads to increased cell death under inflammatory and apoptotic stimuli. Up to 8% of the p53 Pro142 became hydroxylated under the experimental conditions used in our study. This value is close to endogenous hydroxylation levels of certain asparagines in ankyrin repeat domain-containing proteins hydroxylated by the HIF asparaginyl hydroxylase^65,66^ and the centrosomal protein Cep192 hydroxylated by HIF-P4H-1^67^, and may reflect subtle but precise fine-tuning required for the intricate interplay between HIF1α and p53 pathways under various adverse stimuli.

Altogether, our data suggest that HIF-P4H-1 is a common regulator of the HIF1α, NF-κB and p53 pathways. HIF-P4Hs are considered as promising drug targets to intervene in diseases characterized by acute or chronic hypoxia, ranging from severe anemia to tissue ischemia and inflammatory diseases. In addition to inflammation, p53 and NF-κB pathways are regulated in opposite directions in tumors. Therefore, selective HIF-P4H-1 inhibitors may offer a novel way to suppress NF-κB pathway and activate p53 pathway, which could have important implications in the treatment of inflammatory diseases and cancer. However, the interplay between inflammatory and apoptotic pathways is highly complex, and the exact mechanistic roles of HIF-P4H-1 in their regulation requires further studies. Despite the appearing consensus that HIF-P4H-1 inactivation results in protective effects under various inflammatory stimuli, opposing effects on apoptosis have been observed. In contrast to our observations of increased apoptosis, decreased epithelial apoptosis has been observed in a mouse colitis model upon chemical inhibition of HIF-P4Hs or a genetic inhibition of HIF-P4H-1^20,22^. Decreased apoptosis upon inactivation of HIF-P4H-1 has also been observed in *ex vivo* myocardial and *in vivo* liver ischemia/reperfusion injury models and in an *in vitro* neuronal oxytosis model^68,69,70^. Therefore, whether pro- or anti-apoptotic functions of HIF-P4H-1 are dependent on the cell type studied or the severity of the injury model used, needs further clarification.

## Methods

### Generation of *Hif-p4h-1*^−/−^ mice

The targeting construct to inactivate mouse *Hif-ph4-1* is shown in supplementary Fig. 1a and the outcome of successful targeting in supplementary Fig. 1c-f. The deleted exon 3 codes for two catalytically essential amino acids (H309 and D311) that are required for Fe^2+^ binding (supplementary Fig. 1b). The 5′ and 3′ arms of the targeting construct were amplified from C57BL/6 genomic DNA, sequenced and cloned to pBlueScript vector with NeoR cassette (supplementary Fig. 1a). The targeting construct was electroporated into mouse embryonic stem (ES) cells (PRX-B6N #1, derived from C57BL/6N-tac mice) and correctly targeted ES clones were identified by using Southern blotting of genomic DNA (supplementary Fig. 1c). Genotypes of the mice were analyzed by using PCR primers Hif-p4h-1del3ex5F (GACTCAAGGAGCCTCCATATCTC) and Hif-p4h-1del3ex6R (CAACATCAACCTTAACCCACAGTGTAC) for the wt, and loxPT3neoR1 (GCTATACGAAGTTATTAGGTCC) and Hif-p4h-1del3ex4R (CCTTACCTGCCCTAACTGTATGTG) for the targeted allele, producing 2.2-kb and 1.7-kb PCR products, respectively (supplementary Fig. 1d). Deletion of exon 3 (120 bp in size) at the mRNA level was confirmed by PCR analysis of cDNA using primers Hif-p4h-1qP2ex1F (CACGTGGACGCAGTAATCCG) and Hif-p4h-1qP4ex1R (ATGTTGGCTACCACTGGCCG) (supplementary Fig. 1e). The lack of functional HIF-P4H-1 was confirmed also by Western blotting with a HIF-P4H-1 antibody (TA337938, Origene) that recognizes specifically an epitope coded by exon 3 (supplementary Fig. 1f). Before experiments the mice were backcrossed at least 10 times to the C57Bl/6J strain. Animal experiments were approved by the Animal Experiment Board of Finland, following the regulations of the EU Directive 86/609/EEC, the European Convention ETS123 and the national legislation of Finland. The recommendations given by the Federation of European Laboratory Animal Science Associations (FELASA) and the Finnish and EU legislations concerning laboratory animal experiments and handling were followed.

### Cell culture, plasmid and siRNA transfections, and cell treatments

*Hif-p4h-1* ^−/−^ and wt gender matched MEFs were isolated at embryonic day 14.5^71^. Peritoneal macrophages were isolated from 1-year-old female *Hif-p4h-1* ^−/−^ and wt mice as described previously^72^. The human embryonic kidney HEK293 and human breast adenocarcinoma MDA-MB-231 cells were purchased from ATCC. The MEFs, macrophages, HEK293 and *HIF1A* ^−/−^ and wt HCT116 human colon carcinoma cells were maintained in DMEM medium (Invitrogen), and MDA-MB-231 cells in RPMI-1640 medium (Thermo Scientific). The *HIF1A* ^−/−^ and wt HCT116 cells were a gift from Dr. P. Koivunen, University of Oulu. All cell culture media were supplemented with 10% fetal bovine serum (Gibco), 2 mM L-glutamine (Sigma) and 1% penicillin/streptomycin (Gibco). Cell lines used in this study were not authenticated and were tested to be free of mycoplasma contamination. Cell lines used in these experiments were not found in the database of commonly misidentified cell lines that is maintained by ICLAC and NCBI biosample and according to the ATCC the p53 status of HEK293 and HCT116 cells is wild type. The cells were cultured at 37 °C either in 21% oxygen and 5% CO_2_ (normoxia) or exposed to 1% O_2_ (hypoxia) balanced with 5% CO_2_ and 95% N_2_ in an InVivo2 400 hypoxia work station (Ruskinn Technologies).

A mammalian pcDNA expression plasmid encoding recombinant human HIF-P4H-1 with a C-terminal V5His-tag was generated as described for FlagHis-tagged HIF-P4Hs^51^. The pcDNA3-Flag-p53 expression vector was obtained from Addgene (#10838) and the p53 point mutations Pro142Ala and Pro12Ala/Pro13Ala were generated by the Quickchange site-directed mutagenesis kit (Stratagene) according to the manufacturer’s instructions. The p53Δ75-207 deletion construct was a gift from Prof. P. Koivunen, University of Oulu. Plasmid transfections were performed using FuGENE® HD (Promega) or Lipofectamine LTX (Life Technologies) transfection reagents according to the manufacturer’s protocol. Transfection of HIF-P4H-1 and control (scrambled) siRNA oligonucleotides (Santa Cruz Biotechnology, sc-45616 and sc-37007, respectively) was performed with a siRNA transfection reagent (Santa Cruz Biotechnology) according to the manufacturer’s protocol for 24 h. The HIF-P4H-1 knockdown efficiencies are given in supplementary Fig. 2.

The cells were treated with LPS (Sigma), proteasome inhibitor MG132 (Calbiochem), a pan HIF-P4H inhibitor FG4497 (FibroGen), the MDM2 inhibitor nutlin-3a (Calbiochem), staurosporine (Sigma), cisplatin (Santa Cruz), PFTα (Sigma) and cycloheximide (Sigma). The treatment times and doses used before cell harvesting and preparation of the samples are described in the figure legends of the experiments.

### Microarray analysis

MEFs were plated in 6-cm^2^ plates (5 × 10^5^ cells/plate) and cultured in normoxia or hypoxia for 24 h. At the time of harvest the plates were 85% confluent. Two individual primary MEF isolates were prepared from both genotypes and subjected for microarray analysis. Total RNA was extracted using E.Z.N.A. Total RNA Kit 1 (Omega Biotek) according to the manufacturer’s instructions. cDNA was synthesized from 100 ng total RNA and aRNA was generated using the 3′ IVT Express Kit (Affymetrix) according to the manufacturer’s protocol. After fragmentation 10 µg of aRNA was hybridized with the mouse Mouse 430 2.0 GeneChips™ (Affymetrix) following the manufacturer’s protocol and the GeneChips were scanned with an Affymetrix gene chip scanner 3000 7G in Biocenter Oulu DNA Analysis Core Facility.

Affymetrix CEL files (Accession number GSE83535, https://www.ncbi.nlm.nih.gov/geo/) were analyzed using dChip_2010_01 and Chipster v3.4 (http://chipster.csc.fi/) softwares. Array probe data were normalized to the mean expression level of each probe across the samples or data were normalized using the Robust Multiarray Average (RMA) algorithm with variance stabilization. Changes in gene expression between the sample groups were determined using the following criteria. Differentially expressed genes between wt (baseline [B]) and *Hif-p4h-1* ^−/−^ (experimental [E]) MEFs in normoxia and hypoxia were identified by the comparison option of the dChip software. The filtering criteria for this analysis were (a) fold change E/B or B/E ≥ 1.5, (b) lower confidence bound of fold change E-B or B-E = 100, (c) p value for paired t-test < 0.05. All filtering criteria (a)–(c) had to be met for differential gene expression to be included as a positive result. Differently expressed genes were then annotated using hierarchical clustering and gene function enrichment option of the dChip software. Functional classification of genes were further verified by DAVID Bioinformatics Resources. Heatmap for visualizing the grouping of genes was generated using the Chipster software.

### Quantitative real-time PCR (qRT-PCR)

Total RNA was extracted using E.Z.N.A. Total RNA Kit 1 (Omega Biotek) and 1 μg RNA was reverse transcribed into cDNA by using iScript cDNA synthesis kit (Bio-Rad) according to manufecturer’s protocol. The cDNA was diluted 10-fold and qRT-PCR analysis was performed using iTaq Universal SYBR Green Supermix (Bio-Rad). Gene expression levels were analysed in a CFX96 Real-Time PCR Detection System (Bio-Rad) and normalized based on β-actin mRNA expression. Primer sequences used in qRT-PCR are indicated in supplementary Table 4.

### ELISA

*Hif-p4h-1* ^−/−^ and wt MEFs were treated with 200 ng/ml LPS for 24 h. The culture media were collected and centrifuged for 5 mins at 2000 x g and subjected for IL-6, TNF-α and G-CSF analysis by Mouse Inflammatory Cytokines multi-analyte ELISArray kit (Qiagen) according to the manufacturer’s instructions.

### Western blot analysis

Whole cell extracts were prepared using RIPA buffer (50 mM Tris-HCl pH 7.4, 150 mM NaCl, 1% NP-40, 0.5% sodium deoxycholate, 0.1% sodium dodecyl sulfate (SDS)) containing phosphatase (PhosSTOP, Roche) and protease (cOmplete Protease Inhibitor - EDTA Cocktail, Roche) inhibitors. Nuclear and cytoplasmic fractions were prepared using the NE-PER Nuclear and Cytoplasmic Extraction Reagents kit (Thermo Fisher Scientific) according to the manufacturer’s protocol. The extracts were resolved by SDS-PAGE, transferred to polyvinylidene fluoride membranes and probed with the indicated antibodies. The primary antibodies and dilutions used in Western blotting were anti-RelA (MAB3026 1:1000, Millipore), anti-p-Rel-A (Ser 536, 3033 1:500, Cell Signaling), anti-histone H3 (ab18521 1:1000, Abcam), anti-NF-κB p105/p50 (E381 1:1000, Millipore), anti-caspase-3 (9622 1:500, Cell Signaling), anti-cleaved caspase-3 (Asp175 1:200, Cell Signaling), anti-p53 (CM5 1:500, Vector Laboratories, or PAb421 1:2000, Calbiochem, or DO-1 1:2000, Santa Cruz Biotechnology), anti-MDM2 (NBP2-17247 1:1000, Novus Biologicals), anti-HIF1α (NB100-105 1:500, Novus Biologicals), anti-HIF-P4H-1 (BL525 1:500, Bethyl Laboratories, or TA337938 1:300, Origene), anti-V5 (1:500, Life Technologies), anti-Flag M2 (1:1000, Sigma), anti-HA (SAB1305536 1:500 Sigma), anti-hydroxyproline (ab37067 1:200, Abcam), anti-phospho-p53 Ser15 (9284 1:500, Cell Signaling), anti-β-actin (NB600-501 1:20 000, Novus Biologicals) and anti-α-tubulin (T6199 1:20 0000, Sigma), and the secondary antibodies goat anti-rabbit horse radish peroxidase (HRP)-conjugate (1:20 000 Dako) and goat anti-mouse HRP (1:20 000, Dako). Enhanced chemiluminescence (Pierce™ ECL Western Blotting Substrate) was used for detection.

### Ubiquitination assays

For ubiquitination assays, HEK293 cells were first transfected with siRNA as above. After 24 h cells were transfected with a plasmid expressing HA tagged ubiquitin for 24 h and treated with 10 μ μM MG132 for 6 h before harvesting the cells. Cells were lysed in 1% SDS and 10 mM *N*-ethylmaleimide (Sigma) and the lysates were diluted ten-fold in RIPA buffer. Cell lysates were sonicated and equal amounts of protein were immunoprecipitated using p53 beads followed by Western blot analysis.

### Luciferase reporter assays

Luciferase reporter assays were performed using the Dual-Luciferase Reporter Assay System (Promega), according to the manufacturer’s instructions. Briefly, cells were transfected with a kB-TATA-luciferase reporter and a control Renilla luciferase reporter driven by a constitutively active thymidine kinase promoter (pRL-TK)^73^. After 48 h of transfection cells were lysed with the Passive Lysis Buffer (Promega) according to the manufacturer’s instructions. Renilla luciferase activity was used for normalization of the kB-TATA luciferase activity.

### Cell viability assays

To assess caspase 3/7 activity, *Hif-p4h-1* ^−/−^ and wt MEFs and *Hif-p4h-1* ^−/−^ MEFs transfected with empty vector or a vector encoding V5-tagged human recombinant HIF-P4H-1 were seeded in 96-well plates at a density of 20 000 cells/well in triplicates and treated with 100 ng/ml LPS for 24 h. Caspase 3/7 activity was assayed using the Caspase-Glo3/7 Assay (Promega) according to manufacturer’s instructions. To study LPS, staurosporine or cisplatin-induced cell death, the above MEFs were treated with 5 mg/ml LPS for 48 h, 2 μM staurosporine for 24 h or 50 µg/ml cisplatin for 24 h. The MEFs were plated in 6-well plates at a density of 200 000 cells/well, after the treatment washed with 0.15 M NaCl and 0.02 M phosphate, pH 7.4 (phosphate buffered saline, PBS), stained with a saturating concentration of 7-actinomycin D (7-AAD) and analysed by fluorescence-activated cell sorting (FACSCalibur, BD Biosciences). Alternatively, the cells were plated in 96-well plates at a density of 50 000 cells/well, after treatment stained with Live-Dead Cell Staining Kit (BioVision) and viability was determined by dynamic imaging using relative red (dead) and green (live) object count per well in IncuCyteTM zoom live-cell imaging system (Essen BioScience) according to the manufacturer’s instructions.

### Co-immunoprecipitation

The proteasomal inhibitor MG132 (10 μM) was added to cells 4 h before cell lysis to increase the amount of p53 by inhibiting its proteasomal degradation. For co-immunoprecipitation studies cells were lysed in RIPA buffer. 500 µg of the lysate was pre-cleared with 30 µl of Protein A/G plus agarose beads (Santa Cruz Biotechnologies) followed by addition of 3 µl of anti-p53 (PAb421, Calbiochem) or 3 µl of anti-V5 antibodies and incubation for 2 h at 4 °C. 30 µl of Protein A/G plus agarose beads were then added to the antibody conjugated lysate and incubated for 1 h at 4 °C, followed by washing of the beads with RIPA buffer for three times. Proteins bound to the beads were eluted by the soft elution protocol^74^, separated by SDS-PAGE and probed with antibodies indicated in the figure legends.

### Prolyl-4-hydroxylase activity assays, and immunoprecipitation for liquid chromatography-mass spectrometry and for radiochemical and Western blot analysis of 4-hydroxyproline

The activity of recombinant Flag-tagged human HIF-P4H-1 produced in and purified from insect cells was assayed by a method based on measurement of the hydroxylation-coupled stoichiometric release of ^14^CO_2_ from 2-oxo[1-^14^C]glutarate^48,50,51^ using synthetic TCPVQLWVDSTPPPGTR and RCSDSDLAPPQHLIRVEG p53 peptides (Innovagen) and a DLDLEMLAPYIPMDDDFQL HIF1α peptide (Innovagen) as a substrate. In additional experiments HIF-P4H-1 activity was assayed by using L-[2,3,4,5-^3^H]proline (Perkin Elmer) labeled full-length p53 produced with a TNT® Quick Coupled Transcription/Translation System (Promega) as a substrate and the amount of 4-hydroxy[^3^H]proline formed was measured by a radiochemical determination method^50^.

For liquid chromatography-mass spectrometry (LC-MS) overexpressed Flag-tagged p53 was immunoprecipitated from HCT-116 cells treated with 10 μg/ml cisplatin and 10 μM MG132 for 6 h. The cells were lysed in RIPA buffer and the cell lysate was pre-cleared with Protein A/G plus agarose beads (Santa Cruz Biotechnologies). Endogenous p53 was immunoprecipitated with of p53-trap_A beads (50 μl/1 mg cell lysate) (Chromotek) and Flag-tagged p53 with anti-Flag M2 affinity gel (Sigma Aldrich) (10 μl/1 mg cell lysate) by incubation for 1–2 h at 4 °C followed by washing three times with RIPA buffer. The bound proteins were eluted by boiling the p53-trap_A beads in SDS-PAGE sample buffer and by incubating the anti-Flag M2 affinity gel with 150 ng/µl of Flag peptide (Sigma Aldrich) for 45 min at 4 °C. The eluted samples were ran on an SDS-PAGE for LC-MS analysis (see below).

For radiochemical assay of 4-hydroxyproline Flag-tagged p53 was expressed in HCT-116 cells treated with 10 μg/ml cisplatin and 10 μM MG132 with or without 50 μM FG4497 for 6 h in the presence of [2,3,4,5-^3^H] proline (25 μCi/ml). Flag-p53 was immunoprecipitated as described above and the amount of 4-hydroxy[^3^H]proline formed was measured by a radiochemical determination method^50^.

For Western blot analysis of hydroxyproline in Flag-tagged recombinant and endogenous p53, Flag-tagged wt and Pro142Ala p53 were overexpressed in wt HCT-116 cells treated with 10 μg/ml cisplatin and 10 μM MG132 for 6 h, and wt and *HIF1A* ^−/−^ HCT-116 cells were treated with 10 μg/ml cisplatin and 10 μM MG132 for 6 h with or without 50 μM FG4497, respectively. Flag-tagged and endogenous p53 were immunoprecipitated as described above and analysed by Western blotting.

### Protein analysis by mass spectrometry

The area of the SDS gel containing p53 was cut into 1-mm stripes which were individually processed followingly: 3 × 5 min washing steps with 50 mM ammonium-bicarbonate in 40% acetonitrile/60% water to destain the gel, 5 min washing with trypsin buffer (40 mM ammonium-bicarbonate in 9% acetonitrile/91% water) after which 5 μl of trypsin solution (20 ng/μl Sigma proteomics grade trypsin in trypsin buffer) was added to the gel pieces and incubated for 20 min, followed by addition of 15 μl of trypsin buffer. The samples were incubated overnight at 35 °C and the supernatant was transferred to a sample vial before the gel piece was extracted a second time with 50 μl of 0.1% trifluoroacetic acid (TFA) in 30% acetonitrile/70% water. The combined extracts were dried in a speedvac and peptides dissolved in 20 μl of 0.2% TFA. 5 or 10 μl of the solution were subjected to LC-MS analysis using a nano Aquity UPLC system connected to a Synapt G2 Q-Tof mass spectrometer (Waters). Peptides were trapped on a Symmetry C18 0.18 × 20 mM trap column (Waters) for 5 min at a flow of 5 μl/min before separation on a Pico Frit 0.075 × 150 mM column (New Objective) filled with Aquity BEH C18 1.7 μm material (Waters). The pump was operated with a linar gradient from 3 to 40% in 80min, flow 0.3 μl/min (A: 0.1% formic acid in water, B: 0.1% formic acid in acetonitrile). The mass spectrometer was operated between m/z 100 to 1200 in MSE mode, which is a data independent acquisition method without precursor ion selection but continuous switching between high (collision energy ramp 18 to 40 V) and low energy scans (scan time 0.5 sec). Data were processed with PLGS 2.5 Apex 3D (Waters) using automatic peak width and mass tolerance determination, intensity threshold 500, and 5 and 10 counts for elevated and low energy threshold, respectively. Processed spectra were used to search the swissprot database (human) allowing a 4% false positive rate. The search was carried allowing for optional phosphorylation (S,T,Y), N-terminal acetylation, deamidation of Asn and Gln, and oxidation of Pro, Trp and Met. Detailed information of all found fragment ions are provided in Supplementary Tables 5 and 6 and experimental data from PLGS for MS signals matched to p53 peptides in Supplementary Table 7.

### Topical application of 12-O-tetradecanoylphorbol-13-acetate (TPA)

The dorsal skins of male wt and *Hif-p4h-1* ^−/−^ mice were shaved before topical application of 12-O-tetradecanoylphorbol-13-acetate^52^ (TPA, Sigma-Aldrich). 5 μg of TPA was dissolved in 200 μL of acetone and applied topically to the shaved dorsal skin area of each mouse. Nontreated and mice treated only with acetone served as controls. The mice were sacrificed after 24 h and skin biopsies were obtained for histological examination and mRNA isolation. Both the wt and the *Hif-p4h-1* ^−/−^ mice were randomly divided into three groups (untreated, acetone-treated and TPA-treated groups). The age of the mice used in the experiments was 4–6 months.

### Morphometric analysis

Skin samples were fixed overnight in 10% buffered formalin and embedded in paraffin. Sections were stained with hematoxylin and eosin (H&E). Immunohistochemical analysis of macrophages was performed by using a rat anti-mouse Mac-3 antibody (550292 1:50, BD Pharmingen). TUNEL immunofluorescent staining was performed using an *In Situ* Cell death Detection kit, Fluorescein (Roche). Digital images were captured from the desired regions of the skin and morphometric analyses were performed by using Adobe Photoshop CS software. Morphometric analysis was performed from 4–5 sections/mouse and 3–14 mice/genotype as indicated in the figure legend. The investigator was not blinded during the experiment or when assessing the outcome. The *Hif-p4h-1* ^−/−^ mice were analyzed with littermate controls.

### Statistical analysis

Statistical analyses were performed with the Student’s two-tailed or paired *t-*test or Mann-Whitney U-test as indicated in the figure legends. Normal distribution of the data was determined with D’Agostino-Pearson *K* ^2^ *-* test and F-test was used to test whether variances of the samples were equal or not. According to the results from the *K* ^*2*^ and F-tests, appropriate statistical test for obtaining the *P* values was selected. The data are shown as means ± standard deviation (s.d.). *P* values of < 0.05 were considered statistically significant (symbols in figures: *P < 0.05; **P < 0.01; ***P < 0.001).

### Data availability

Materials, data and associated protocols will be promptly available. The *Hif-p4h-1* ^−/−^ mouse line will be available under a material transfer agreement.

## Electronic supplementary material

Suppl Figs 1-5 and Suppl Tables 1-4

Dataset 1

Dataset 2

Dataset 3
