# Supplementary material for: Hypoxia-inducible factor prolyl-4-hydroxylase-1 is a convergent point in the reciprocal negative regulation of NF-κB and p53 signaling pathways
Source: Sci Rep. 2017 Dec 8;7:17220. doi: 10.1038/s41598-017-17376-0 (PMC5722952; doi:10.1038/s41598-017-17376-0)
Supplement: Supplementary file 1 — Suppl Figs 1-5 and Suppl Tables 1-4 [file 41598_2017_17376_MOESM1_ESM.pdf]

Hypoxia-inducible factor prolyl-4-hydroxylase-1 is a convergent point in the reciprocal negative regulation of NF-κB and p53 signaling pathways

Karim Ullah[1,2,3], Ann-Helen Rosendahl[1,2,3], Valerio Izzi[1,2,3], Ulrich Bergmann[2], Taina Pihlajaniemi[1,2,3], Joni M. Mäki[1,2,3], Johanna Myllyharju[1,2,3]

[1]Oulu Center for Cell-Matrix Research, [2]Biocenter Oulu and [3]Faculty of Biochemistry and Molecular Medicine, University of Oulu, FIN-90014, Finland

Supplementary data file

Contents:

Legends to supplementary figures 1-5

Supplementary figures 1-5

Supplementary tables 1-4

Supplementary tables 5-7 are uploaded as separate source data files

Supplementary Figure 1. Generation of *Hif-p4h-1*$^{-/-}$ mice. (**a**) Schematic representation of the gene targeting strategy. The organization of the wt gene, the targeting construct, and the structure of the locus following gene targeting are shown. Exons are depicted as gray boxes and numbered. The location of PCR primers and *Spe I* restriction sites used for Southern hybridization with 3'probe for detection of the gene targeting event are shown. (**b**) Schematic representation of the outcome of the in-frame deletion of exon 3 at protein level. The deleted exon 3 codes for two catalytically critical amino acid residues (H309 and D311) required Fe^{2+} binding and thus the deleted protein is an inactive HIF-P4H-1. (**c**) Identification of the targeting in ES cells by using the 3' probe and *Spe I* digestion in Southern blots, where 9.8-kb wt (+) and 5.2-kb *Hif-p4h-1* mutant (-) *Spe I* fragments were identified, as expected. (**d**) Genotyping by using PCR of genomic DNA from F2 generation mice showing 2.2-kb and 1.7-kb bands for wt (+) and *Hif-p4h-1* mutant alleles (-), respectively. (**e**) Deletion of exon 3 was confirmed at the mRNA level by PCR of cDNA isolated from wt and *Hif-p4h-1*$^{-/-}$ mice. PCR primers Hif-p4h-1qP2ex1F and Hif-p4h-1qP4ex1R from exons 2 and 4, respectively, were used to confirm deletion of exon 3. As a result 245-bp wt (+) and 125-bp mutant (-) bands were amplified. (**f**) Western blot analysis of HIF-P4H-1 in wt and *Hif-p4h-1*$^{-/-}$ MEFs.

Supplementary Figure 2. Relative *HIF-P4H-1* mRNA expression in (a) HEK293 and (b) *HIF1A*$^{-/-}$ HCT-116 cells transfected with scrambled or *HIF-P4H-1* siRNA. $^*P<0.05$, $^{**}P<0.01$ and $^{***}P<0.001$, two-tailed Student's t-test.

Supplementary Figure 3. Flag-tagged p53 was immunoprecipitated from HCT-116 cells treated with 10 μg cisplatin and 10 μM MG132 for 6 h, separated by SDS-PAGE, trypsinized and analyzed by LC-MS. Mass signals extracted from the high energy trace of the MS^e measurement assigned to the peptide TCPVQLWVDSTPPPGTR (140-156) are shown. MS/MS spectra of (**a**) the p53 wt peptide 140-156 and (**b**) the Pro142Ala mutant. A detailed list of all found fragment ions is given in supplementary Tables 5 (wt p53) and 6 (Pro142Ala mutant).

Supplementary Figure 4. Lack of HIF-P4H-1 increases the apoptotic activity in mouse skin. Histological analysis of apoptotic cells in the skin stained by TUNEL immunofluorescent staining

using Hoechst (1:10000) stain as counterstaining.

Supplementary Figure 5. Scans of developed films with approximate regions used for figures marked with rectangles and molecular sizes indicated in kDa (based on prestained protein markers).

Suppl. FIGURE 1.

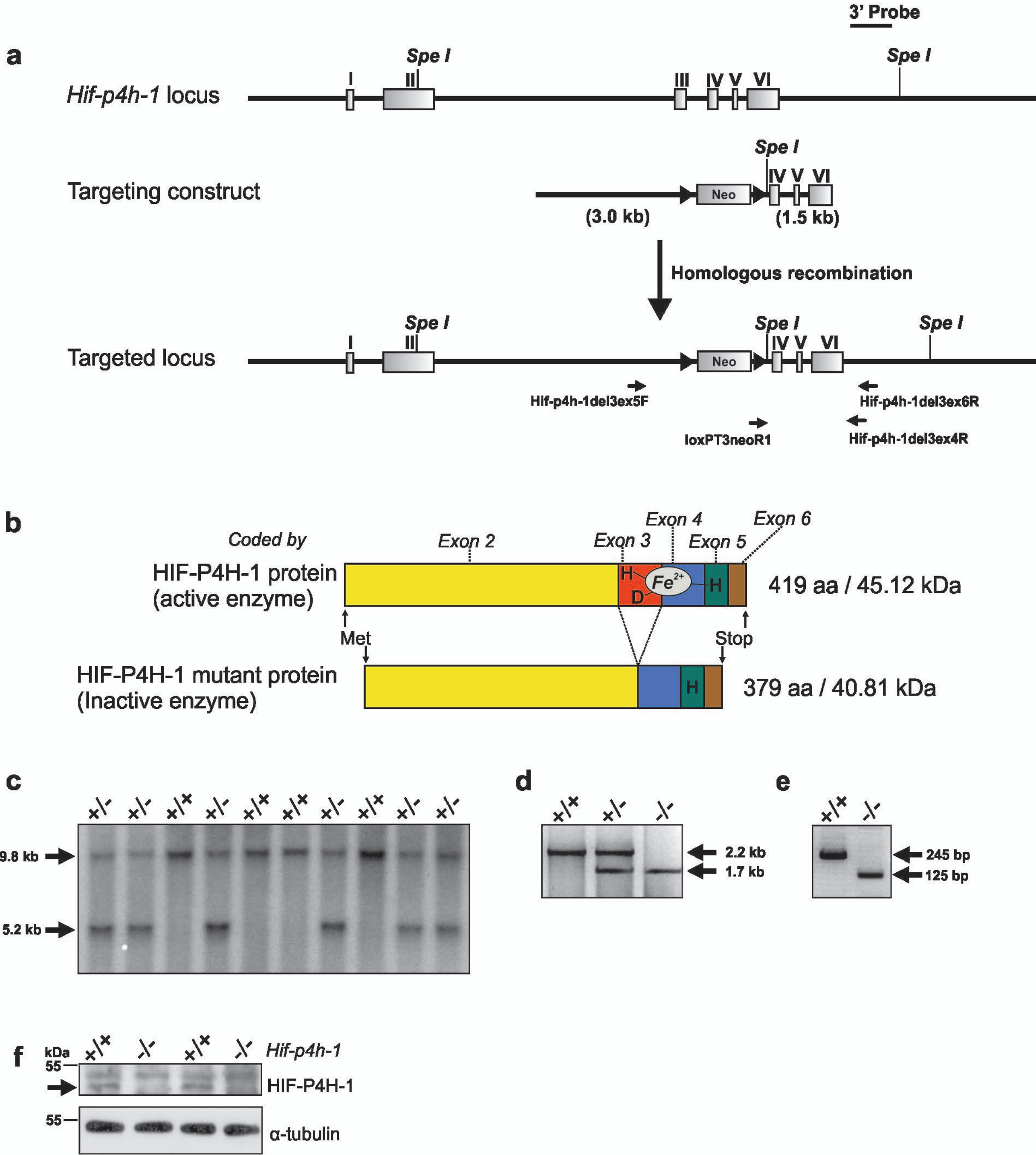

Suppl. FIGURE 2.

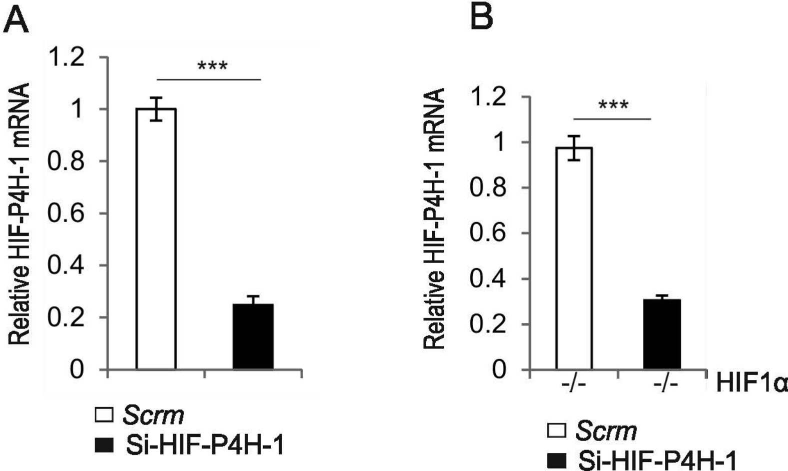

Suppl. FIGURE 3.

A

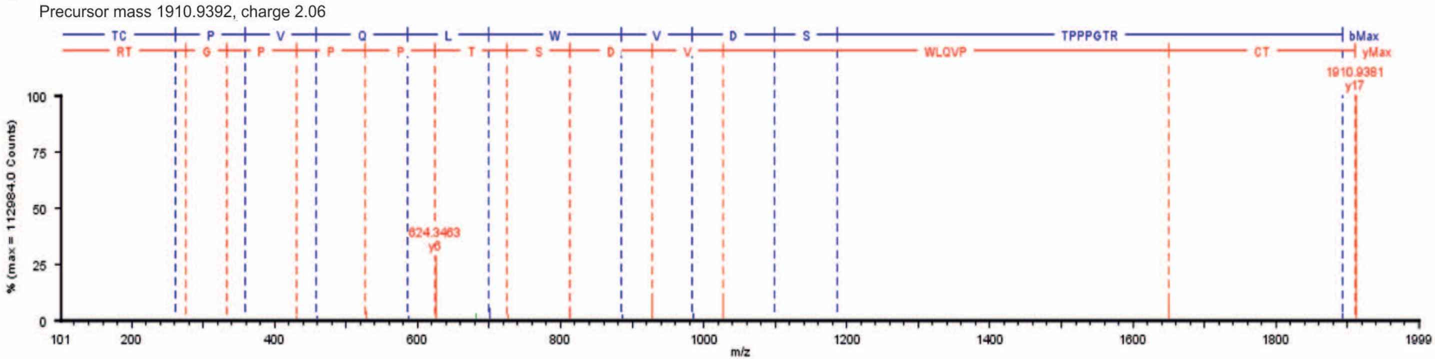

B

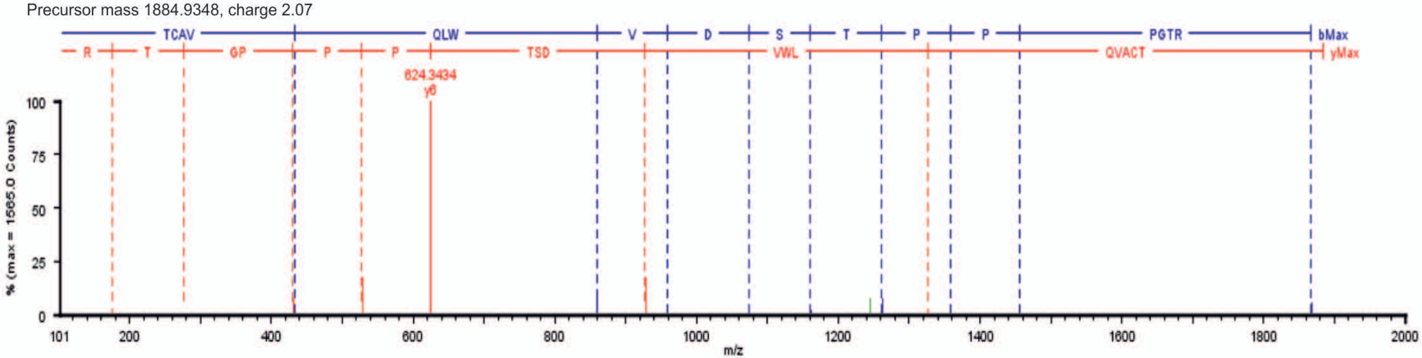

Suppl. FIGURE 4.

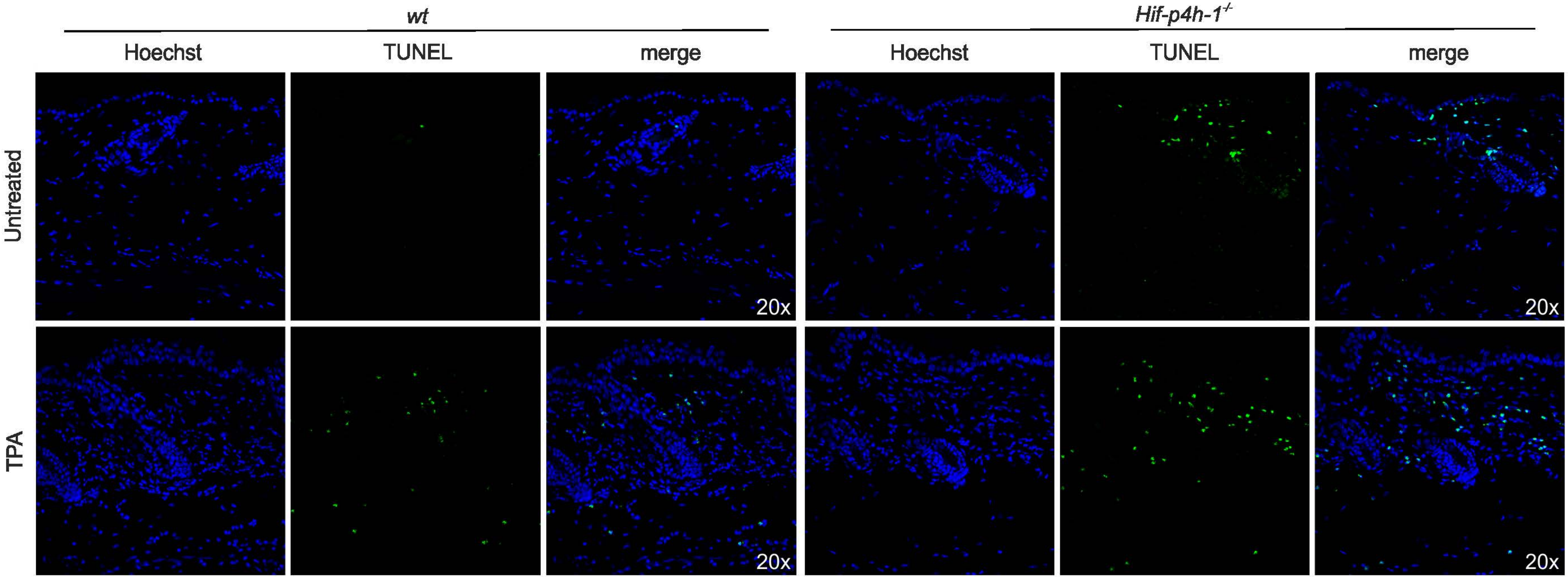

Supplementary Figure 5

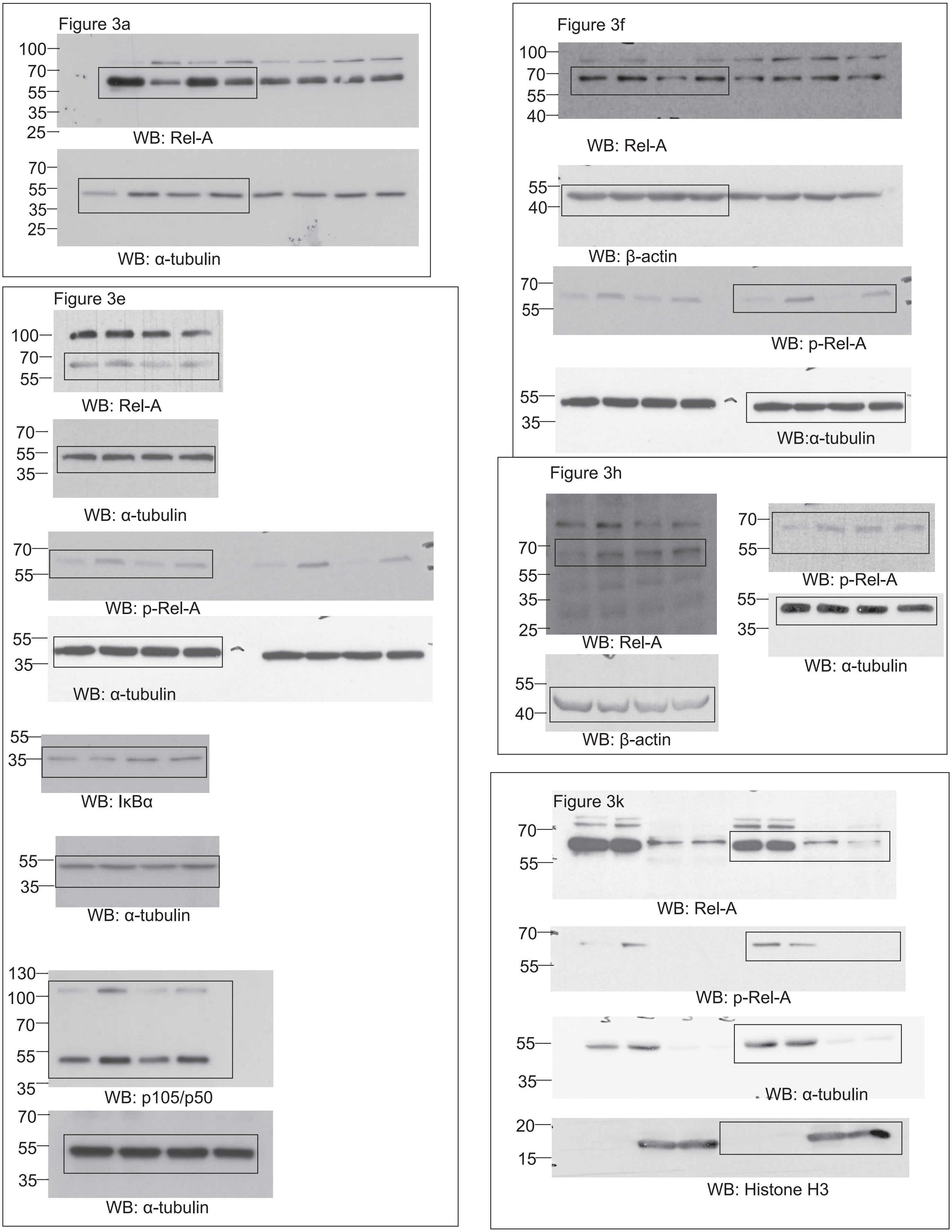

Supplementary Figure 5

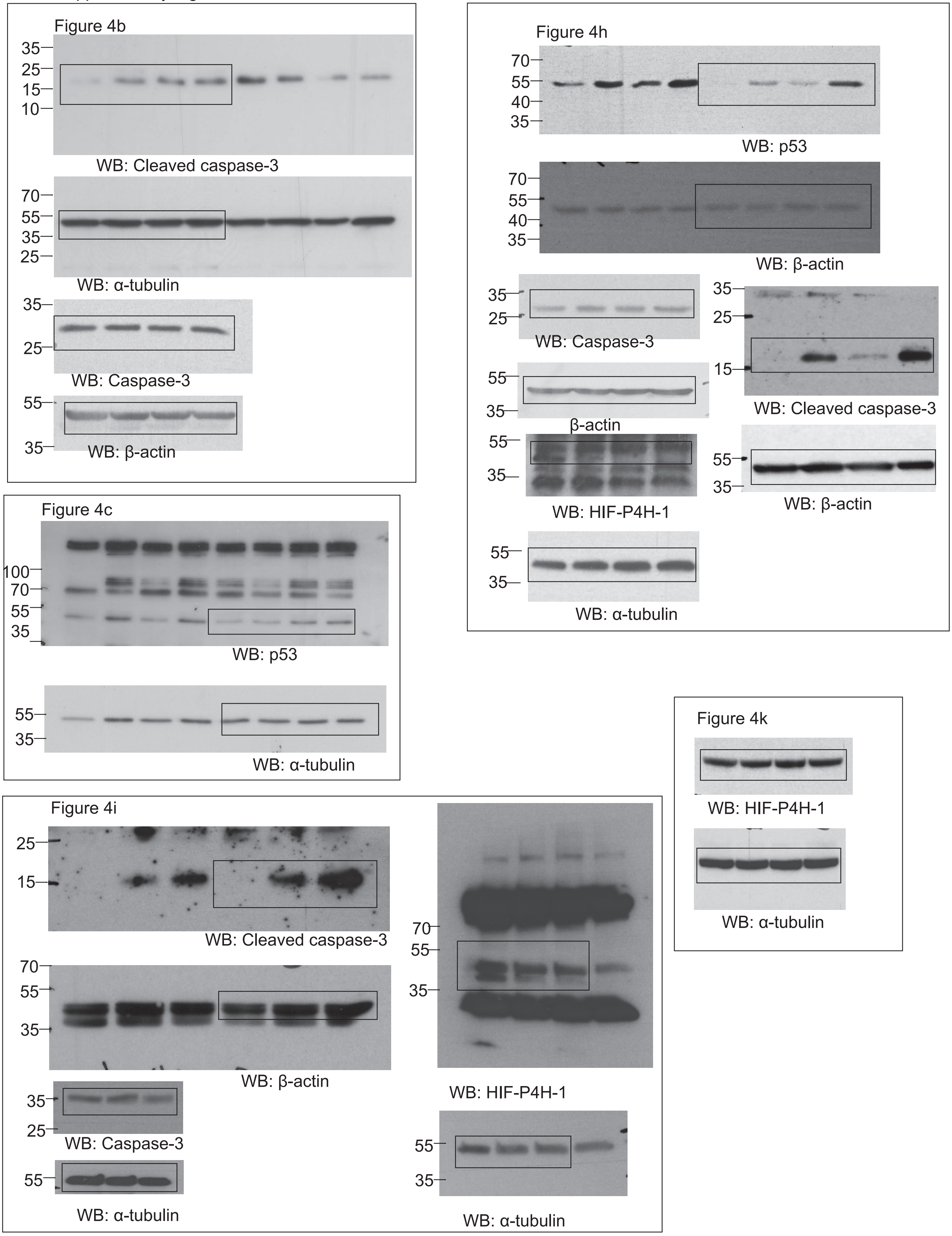

Supplementary Figure 5

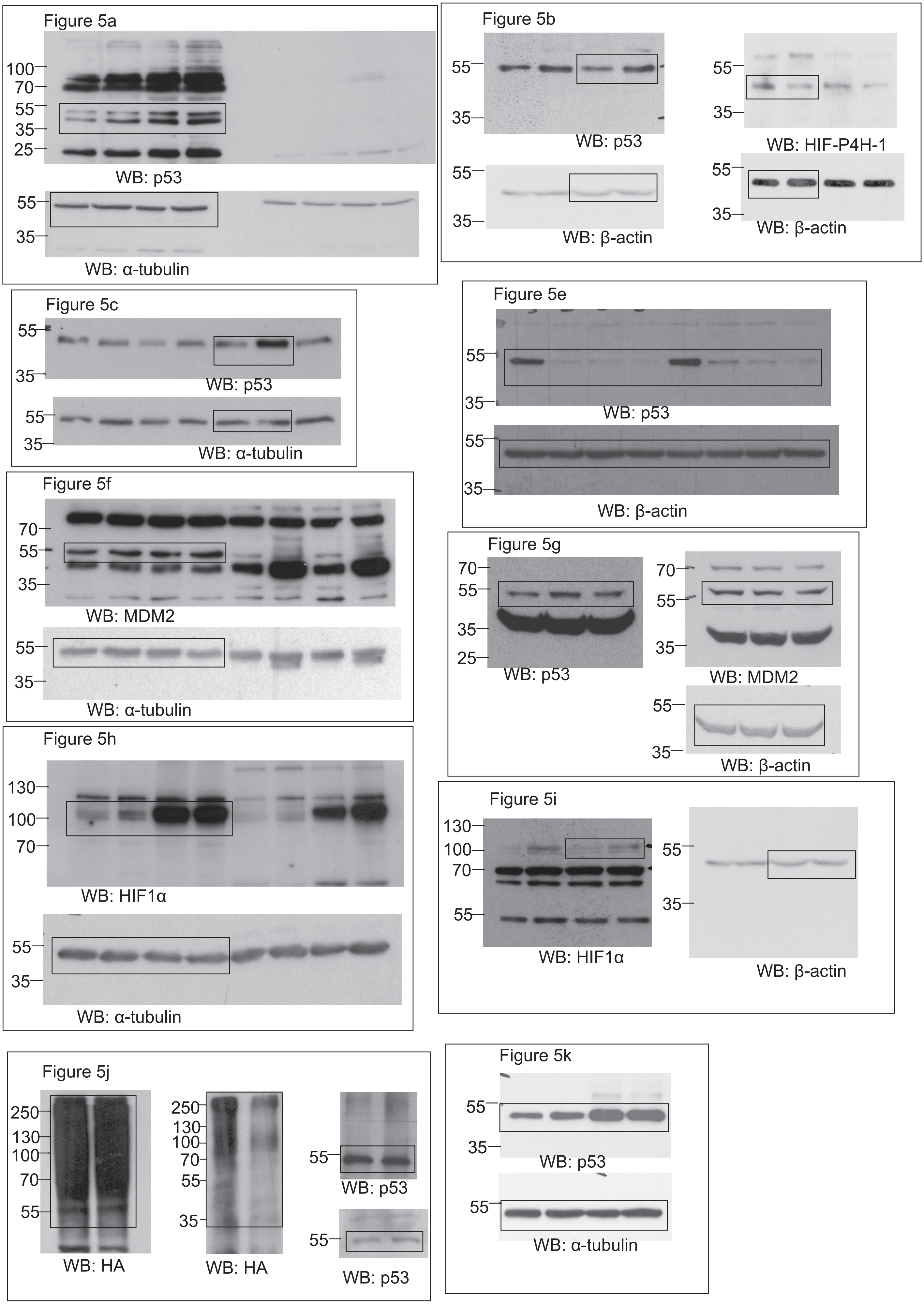

Supplementary Figure 5

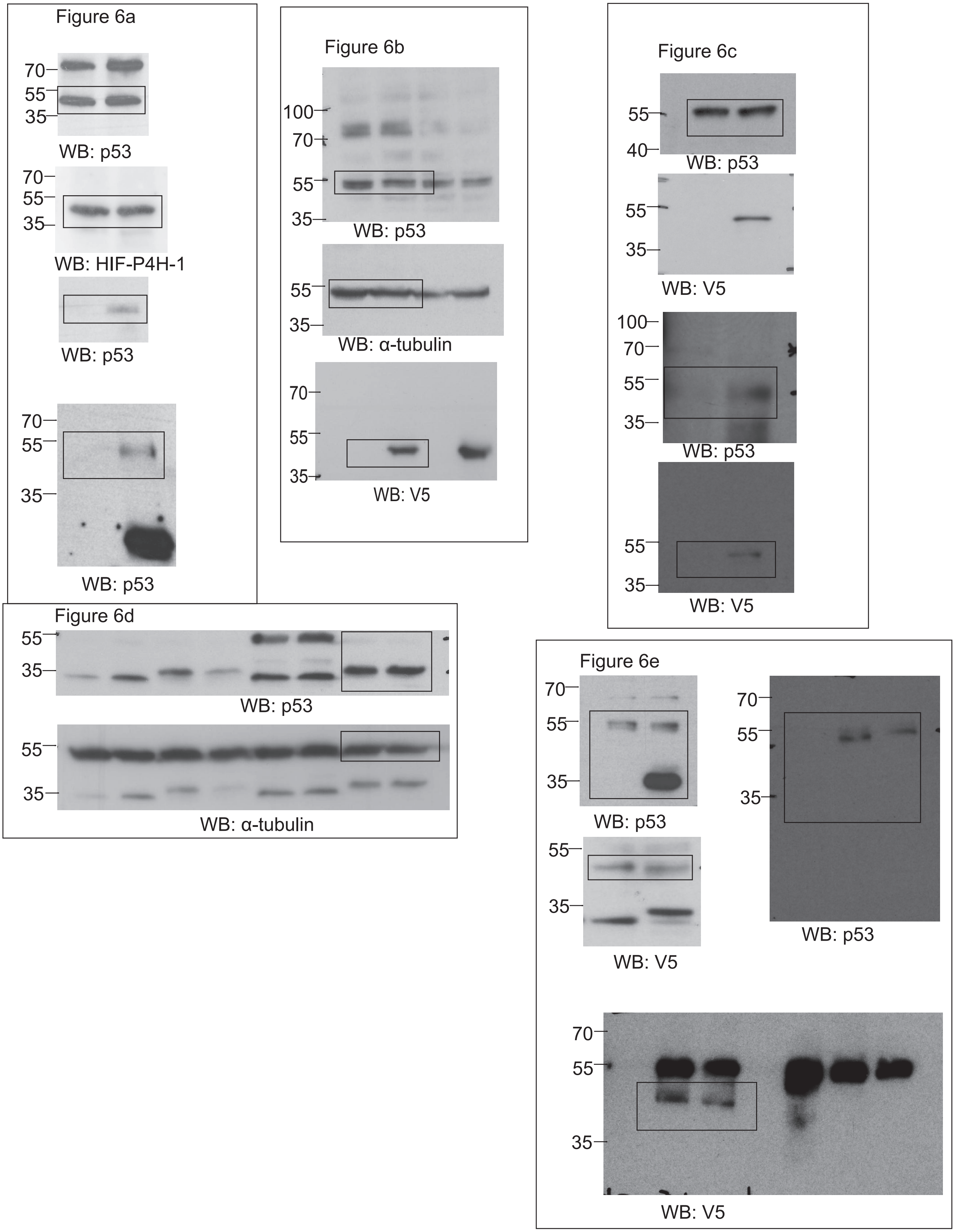

Supplementary Figure 5

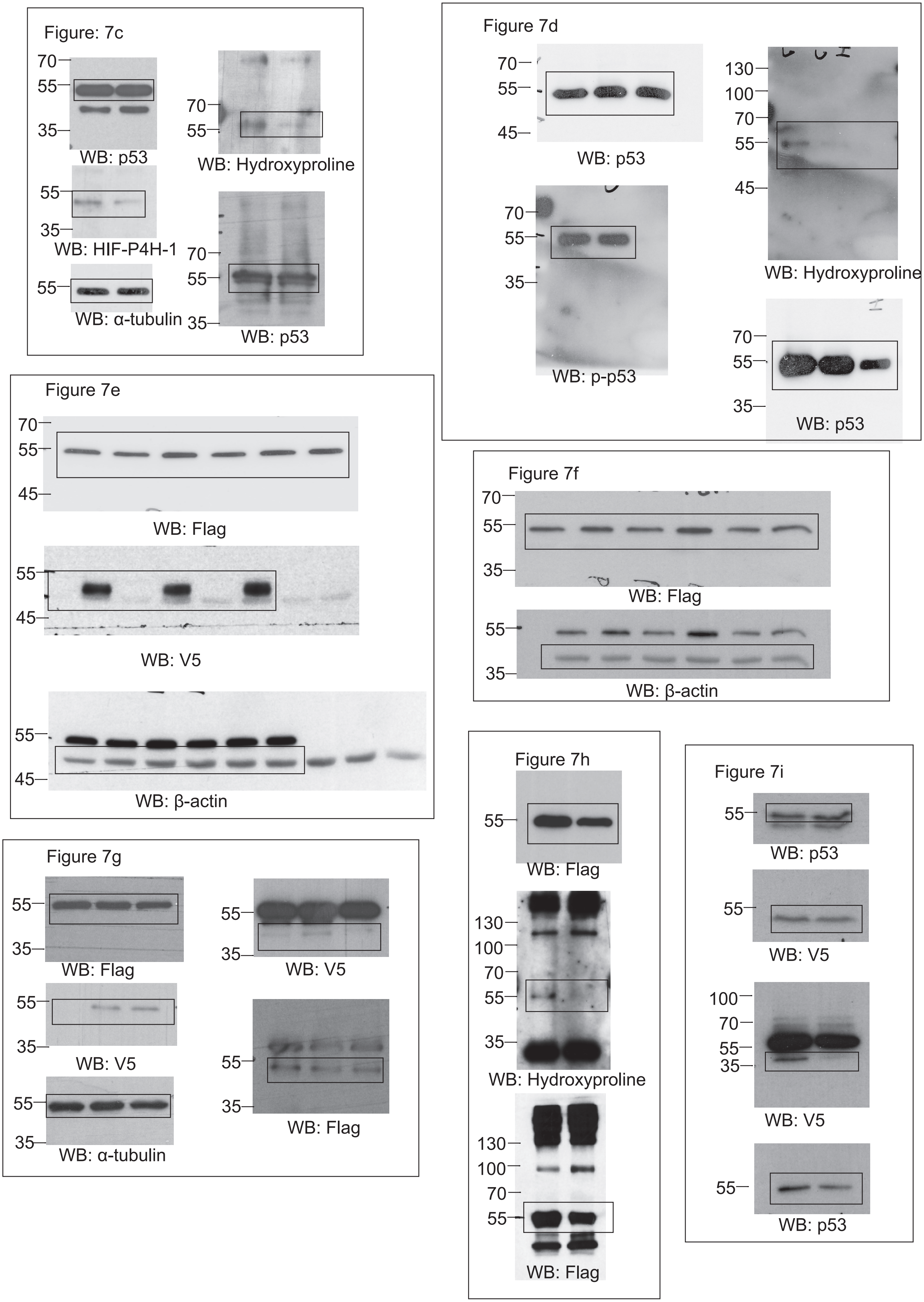

Supplementary Figure 5

Suppl. Figure 1c

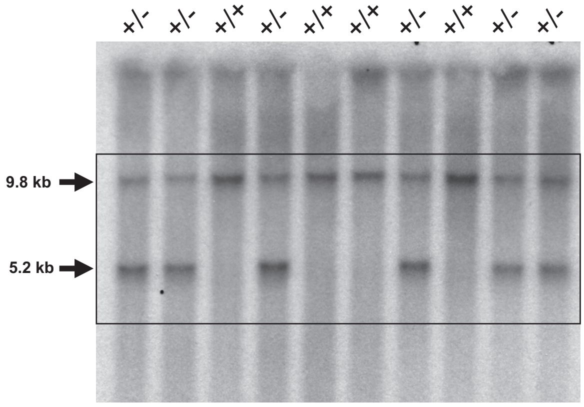

Suppl. Figure 1f

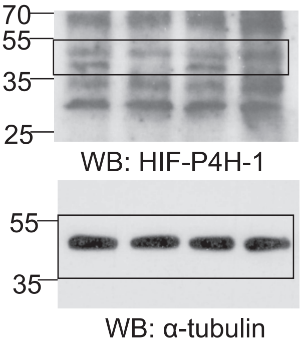

Suppl. Figure 1d

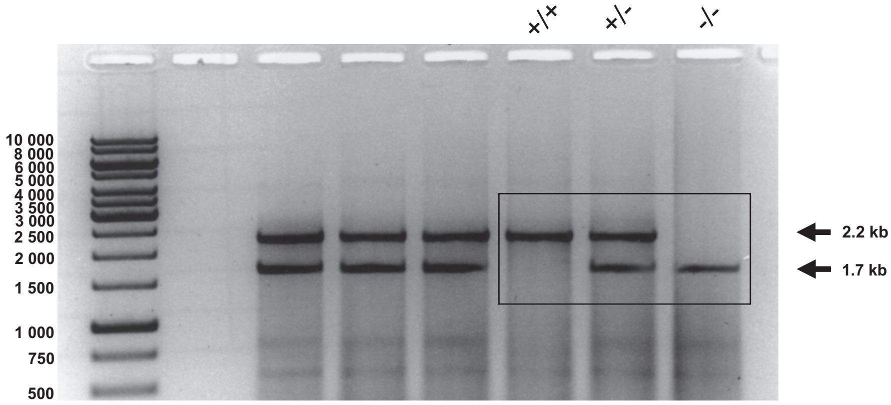

Suppl. Figure 1e

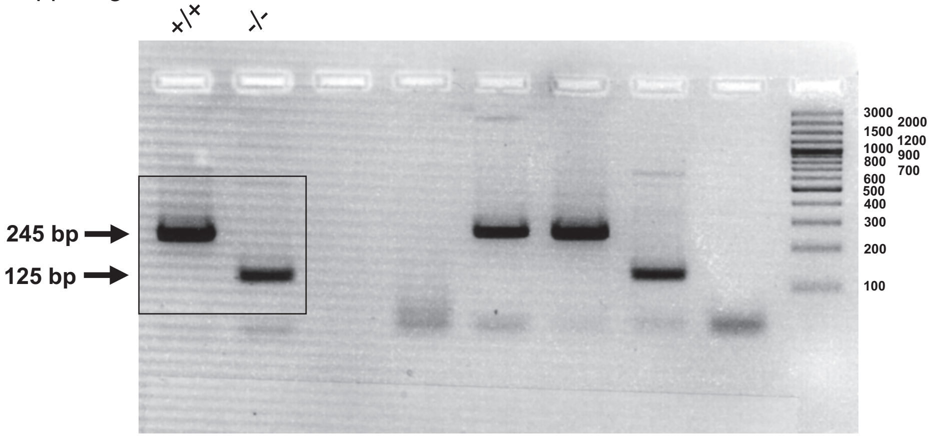

Suppl. Table 1. Inflammatory genes that are downregulated in *Hif-p4h-1*$^{-/-}$ MEFs relative to wt according to microarray analysis. Mean values of two separate microarray analyses of individual MEF isolates are shown.

Gene Name	Accession no.	Fold change *Hif-p4h-1*$^{-/-}$/wt in Normoxia	Fold change *Hif-p4h-1*$^{-/-}$/wt in Hypoxia
C1qc	NM_007574	-6.845	-5.955
Fcgr3	NM_010188	-5.325	-2.475
Fcer1g	NM_010185	-5.115	-3.765
C1qa	NM_007572	-4.465	-5.935
Ctss	NM_021281	-3.87	-3.025
C1qb	BB111335	-2.575	-2.53
Tac1	NM_009311	-2.455	-1.985
C1qb	AW227993	-2.2	-2.315
Ednra	AW558570	-1.745	-0.56
Cxcl10	NM_021274	-1.7	-1.54
Ptgis	NM_008968	-1.635	-1.67
Ctla2a	NM_007796	-1.37	-1.44
Nov	X96585	-1.36	-1.26
S1pr3	AV238324	-1.35	-1.3
Tnfaip6	NM_009398	-1.31	-1.305
Cxcl1	BB554288	-1.195	-1.275
Chst2	NM_018763	-1.19	-1.35
Jmjd3	AW763746	-1.135	-1.245
Cd14	NM_009841	-0.195	-1.395
Ash1l	BG694892	-0.09	-1.205
Igfbp4	BB787243	-0.05	0.085
Tnfrsf1a	L26349	0.035	1.165
Tnfrsf1a	L26349	0.035	1.165
Slit2	BG963150	0.11	0.095
Tgm2	AW321975	0.19	0.2
Serpine1	NM_008871	0.19	0.17
Fabp4	NM_024406	0.74	0.115
Cxcl5	NM_009141	0.765	0.465
Il1rl1	D13695	1.14	1.27
C1qtnf3	NM_030888	1.62	1.43

Suppl. Table 2. Changes in the expression level of apoptotic genes in *Hif-p4h-1*$^{-/-}$ MEFs relative to wt according to microarray analysis. . Mean values of two separate microarray analyses of individual MEF isolates are shown.

Gene Name	Accession no.	Fold change *Hif-p4h-1*$^{-/-}$/wt in Normoxia	Fold change *Hif-p4h-1*$^{-/-}$/wt in Hypoxia
Perp	NM_022032	2.285	2.015
Bnip3	NM_009760	1.775	-1.04
Tnfrsf11b	AB013898	1.55	1.23
Bid	NM_007544	1.4	1.35
Ripk2	NM_138952	1.38	0.325
Sulf1	BB065799	1.34	1.285
Gadd45g	AK007410	1.325	0.225
Pmaip1	NM_021451	1.315	1.505
Hmox1	NM_010442	1.26	1.2
Trp53inp1	AW495711	1.165	1.315
Bcl2l1	NM_009743	1.135	1.22
Aen	BI440638	1.075	1.255
Trp53	BB828014	1.025	1.725
Gadd45b	AK010420	0.185	0.195
App	NM_007471	0.15	1.18
Sgk1	NM_011361	0.15	-0.03
Ppp1r13l	BB752796	0.105	0.165
Bcap31	AV066734	0.055	1.145
Dap	BC024876	0.055	1.2
Tnfrsf12a	NM_013749	0.04	0.115
Tnfrsf1a	L26349	0.035	1.165
Mcl1	BC003839	0.02	1.165
Zmat3	NM_009517	0.015	0.16
Cflar	BC023121	0.01	0.11
Ckap2	BM208103	0.01	1.24
Rad21	AF332085	-0.02	-0.18
Egln3	BB284358	-0.04	-1.19
Cadm1	NM_018770	-0.55	-0.275
Phf17	BG065238	-1.155	-1.18
Tgfbr1	BM248342	-1.185	-1.23
Bcl2l11	BB667581	-1.195	-1.235
Gas2	NM_008087	-1.255	-1.215
Trib3	BB508622	-1.265	-1.12
Peg3	AB003040	-1.295	-1.275

Suppl. Table 3. Changes in the expression level of anti-apoptotic genes in *Hif-p4h-1*$^{-/-}$ MEFs relative to wt according to microarray analysis

Gene name	Accession no.	Fold change *Hif-p4h-1*$^{-/-}$/wt in Normoxia	Fold change *Hif-p4h-1*$^{-/-}$/wt in Hypoxia
Fcer1g	NM_010185	-5.115	-3.765
Krt18	NM_010664	-1.73	-1.525
Kitl	BB815530	-1.23	-1.345
Tgfbr1	BM248342	-1.185	-1.23
Bcl11b	BM117007	-1.175	-0.185
Fgfr1	M33760	-0.13	-1.31
Chst11	AK003880	-0.01	-0.035
Atf5	AF375476	-0.005	-0.085
Cflar	BC023121	0.01	0.11
Btg2	NM_007570	0.065	1.485
Cdkn1a	AK007630	0.075	1.255
Htt	L23312	0.08	1.405
Ercc5	BM198879	0.1	0.21
Grina	AW212189	0.11	0.115
Cacna1a	AB066608	0.2	1.38
Hspa1b	M12573	1.035	1.195
Bcl2l1	NM_009743	1.135	1.22
Spp1	NM_009263	1.145	1.155
Prnp	BE630020	1.2	0.165
Foxc2	NM_013519	1.21	0.23
Bnip3l	AK018668	1.24	-0.02
Hmox1	NM_010442	1.26	1.2
Tgfb3:	BC014690	1.34	1.375
Bdnf	AY057913	1.365	1.275
Vnn1	NM_011704	1.59	0.72
Vegfa	NM_009505	1.59	-0.09
Msx2	NM_013601	1.86	1.95

Suppl. Table 4. Primer sequences used in qPCR analysis.

Target	Direction	Sequence (5'-3')
FCGR3	Forward	5-TCGGTGTCAAATGGAGCAGACC-3
	Reverse	5-CTATGGCACCTTAGCGTGATGG-3
C1QA	Forward	5-GTGGCTGAAGATGTCTGCCGAG-3
	Reverse	5-TTAAAACCTCGGATACCAGTCCG-3
CXCL10	Forward	5-ATCATCCCTGCGAGCCTATCCT-3
	Reverse	5-GACCTTTTTTGGCTAAACGCTTTC-3
C1QC	Forward	5-AAGGACGGGCATGATGGACTCC-3
	Reverse	5-TTTCCCACGGTGGCCAGGCAT-3
PERP	Forward	5-TCCAGACATCGTCGCTTTGGTG-3
	Reverse	5-AGAGAATGAAGCAGATGCACAGG-3
BNIP3	Forward	5-GCTCCAAGAGTTCTCACTGTGAC-3
	Reverse	5-GTTTTTCTCGCCAAAGCTGTGGC-3
IL6	Forward	5-TACCACTTCACAAGTCGGAGGC-3
	Reverse	5-CTGCAAGTGCATCATCGTTGTTC-3
COX2	Forward	5-GCGACATACTCAAGCAGGAGCA-3
	Reverse	5-AGTGGTAACCGCTCAGGTGTTG-3
Mouse TNF A	Forward	5-GGTGCCTATGTCTCAGCCTCTT-3
	Reverse	5-GCCATAGAACTGATGAGAGGGAG-3
Human-TNF-A	Forward	5-CTCTTCTGCCTGCTGCACTTTG-3
	Reverse	5-ATGGGCTACAGGCTTGTCACTC-3
PDK1	Forward	5-CCACTGAGGAAGATCGACAGAC-3
	Reverse	5-AGAGGCGTGATATGGGCAATCC-3
P53	Forward	5-CACGTACTCTCCTCCCCTCA-3
	Reverse	5-AACTGCACAGGGCACGTCTT-3
HIF1α	Forward	5-GGCGAGAACGAGAAGAAAAA-3
	Reverse	5-AAGTGGCAACTGATGAGCAA-3
Human HIF-P4H-1	Forward	5-CTGTCTGGTATTTTGATGCCAAGG-3
	Reverse	5-CGGCTGTGATACAGGTACTTGG-3
Mouse REL A	Forward	5-TGAACCGAAACTCTGGCAGCTG-3
	Reverse	5-CATCAGCTTGCGAAAAGGAGCC-3
